# Microbiome dynamics associated with *Hematodinium* sp. infection in Norway lobster (*Nephrops norvegicus*)

**DOI:** 10.1186/s42523-025-00416-w

**Published:** 2025-06-13

**Authors:** Irene Martin, Ahmed Elsheshtawy, Benjamin Gregory James Clokie, Simon MacKenzie, Kelly Simone Bateman, David Bass, Grant D. Stentiford, Amaya Albalat

**Affiliations:** 1 Institute of Aquaculture, University of Stirling, Stirling, FK9 4LA UK; 2 Faculty of Aquatic and Fisheries Sciences, Kafrelsheikh University, Kafr El Sheikh City, 33516 Egypt; 3 Weymouth Laboratory, Centre for Environment, Fisheries and Aquaculture Science (Cefas), Weymouth, Dorset DT4 8UB UK; 4 Sustainable Aquaculture Futures, Biosciences, College of Life and Environmental Sciences, University of Exeter, Stocker Road, Exeter, UK

**Keywords:** Haemolymph microbiome, Gut microbiome, Dysbiosis, Parasitism, Host-microbiome interaction, Host-parasite interaction, Decapod, Dinoflagellates

## Abstract

**Background:**

The parasite *Hematodinium* sp. causes morbidity and seasonal mortality events in more than 40 decapod species globally and therefore, it is now recognised as a significant threat to the future sustainability of shellfish fisheries and aquaculture worldwide. Among these, Norway lobster (*Nephrops norvegicus*), an important representative of the marine benthos and supporting the most valuable shellfish fishery in the UK, experience yearly seasonal *Hematodinium* sp. patent infections. Currently, little is known about the *N. norvegicus* microbiome and potential role during *Hematodinium* sp. infection. Therefore, in this study we investigated the microbiome dynamics of *N. norvegicus* associated with *Hematodinium* sp. infection and disease progression in the haemolymph and gut. *N. norvegicus* were sampled from the Clyde Sea Area, Scotland during the peak of the *Hematodinium* sp. patent infection. The presence and intensity of *Hematodinium* sp. infection were determined using the body colour method (BCM), pleopod method (PM), histology (heart, gonads, hepatopancreas, gills and muscle) and molecular tools (PCR).

**Results:**

Marked shifts in the bacterial richness of the haemolymph and significant alterations in the overall bacterial community composition of both tissues were observed in infected lobsters. These changes, observed even at subpatent levels of infection (only positive by PCR), indicate a prompt and persistent microbiome shift associated with *Hematodinium* sp. infection. Furthermore, smaller healthy animals (25.2 ± 1.20 mm CL) known to be particularly susceptible to high severity infection displayed a decreased microbiome richness in the haemolymph suggesting a potential link between the host microbiome and susceptibility to disease progression, a possibility that merits further research.

**Conclusions:**

This study offers the first insights into the pathobiome of *N. norvegicus* due to *Hematodinium* sp. infection and disease that in turn provides a foundation for further studies on the pathogenesis of this important parasitic disease.

**Supplementary Information:**

The online version contains supplementary material available at 10.1186/s42523-025-00416-w.

## Introduction

Understanding the underlying mechanisms associated with parasite success is crucial in both fundamental and applied biology and it has major implications in the way we interpret epidemiological studies. It is now well recognised that examining host-parasite interactions as a two-player system is oversimplistic given that both host- and parasite-associated microorganisms may play a role in the phenotypic alterations resulting from parasitism [1, 2]. Parasites may influence the microbiome by acting as vectors or reservoirs of microbes, driving the evolution of defensive microbes, competing with host microbiota or providing metabolic reservoirs to support the growth and survival of other host microbial species [3]. Alternatively, host microbiome shifts linked to the immune status of its host might influence parasite success by making the environment more or less suitable [4]. Therefore, understanding the role of the microbiome within the context of disease also known as pathobiome is crucial to map out parasite strategies for success [2].

Dinoflagellates of the genus *Hematodinium*, which include at least three species, *H.perezi*, *H.australis* and *Hematodinium* sp., have been reported to infect a wide range of crustacean hosts across the globe (> 40) [5–8]. Among host-*Hematodinium* systems, the *Nephrops-Hematodinium* system in the Clyde Sea Area (CSA) on the West Coast of Scotland has been extensively investigated [9–17]. Methods for the detection of the patent infection include a visual examination of the altered carapace coloration (body colour method, BCM) and the pleopod staging method (PM) [18]. While the BCM can only detect advanced levels of infection, the PM is more sensitive and allows to categorise disease progression using a four-point scale based on the aggregation of the parasite within the pleopod. On the other hand, subpatent levels of *Hematodinium* sp. infection can be identified using tissue-based histology and molecular-based methods performed on haemolymph samples[19, 20].

In advanced stages of infection, *N. norvegicus* appear lethargic and moribund due to metabolic exhaustion (e.g. hypoproteinaemia) [21, 22]. A key landmark trait of *Hematodinium* infection is the reported lack of cellular host reactivity [23], which could be associated with the parasite suppressing or evading the host immune system. Currently, there are no data supporting the hypothesis that *Hematodinium* suppresses the host's immune system [24]. Therefore, the lack of cellular host reactivity associated with disease progression could be ascribed to the immune evasion hypothesis, an ubiquitous strategy that can be supported by a plethora of molecular mechanisms [25]. In this regard, a recent study has shown differences in protein deamination/citrullination signatures associated with haemocyte adhesion in *Hematodinium* sp. infected crabs (*Carcinus maenas*) indicating a role for the parasite itself and/or other symbionts in modulating host immune responses via deamination [26]. Several studies have explored the association of microbiome changes in the regulation of decapod health and disease in several species [27–29] including commercially important tropical decapod species, as reviewed by Holt and colleagues [30]. Due to the links between the gut microbiota and the host immune system, the onset of pathogenesis is often associated to a reduction in bacterial diversity in the gut or a differential abundance of specific microbial taxa [31]. In the case of *Hematodinium* infections, one of the key pathological alterations takes place in the haemolymph, where the high number of parasites alters the appearance of the normally translucid haemolymph into a denser, milky/cream-like fluid (see in Small [32]). Traditionally, the haemolymph of healthy invertebrates was considered sterile. However, an increasing number of studies have demonstrated that crustacean haemolymph is not sterile [33, 34] but rather rich in bacterial constituents. A study by Stentiford and colleagues in 2012 also demonstrated that bacterial symbionts may also occur within the cytoplasm of *Hematodinium* cells, infecting shrimp [35].

This study aimed to characterise the haemolymph and gut microbiomes of *N. norvegicus* and investigate if there is a pathobiome associated with *Hematodinium* sp. infection and more specifically disease progression. To this end, *N. norvegicus* were sampled from the CSA during the peak of the *Hematodinium* sp. patent infection in March 2020. The presence and intensity of *Hematodinium* sp. infection were determined using the BCM, PM, histology (heart, gonads, hepatopancreas, gills and muscle) and molecular tools (PCR in haemolymph). The microbiome of both haemolymph and gut were profiled using 16S rRNA amplicon sequencing.

## Materials and methods

### Ethics statement

This study protocol was approved by the University of Stirling Animal Welfare and Ethical Review Body (AWERB (18/19) 39).

### Sample collection

A total of n = 349 *Nephrops norvegicus* were trawled from the Clyde Sea Area (55°45.482 N, 4°53.375 W) aboard the commercial vessel Eilidh Anne GK2 on the 9 th of March 2020. A non-targeted subset of animals consisting of n = 149 *N. norvegicus* was collected and placed on ice. This set, referred to as the ‘prevalence-set’, was used to determine the prevalence of *Hematodinium* sp. infection at that specific time. Another set of n = 200 *N. norvegicus*, classified on-board the vessel based on their visual appearance (targeting BCM positive and negative), were placed in running seawater and transported live to the Field Studies Council (FSC) Millport facilities. Animals from this sub-set were kept in tanks with running seawater and are referred to as the ‘pathobiome-set’. Data collected from the ‘prevalence-set’ included sex, carapace length (CL), and moulting stage. The level of *Hematodinium* sp. infection in the ‘prevalence-set’ was determined by employing the body colour method (BCM) and pleopod method (PM), as per the four-stage criteria established by Field and Appleton [18]. The same information was recorded for animals in the ‘pathobiome-set’. However, in this subset, additional samples were taken for molecular analyses (PCR detection of *Hematodinium* sp. in the haemolymph), microbiome investigation (haemolymph and gut) and histological analysis (heart, gonads, hepatopancreas, gills, and muscle). Haemolymph samples (0.5 mL) were obtained from the base of the fifth pereiopod using a sterile syringe coupled with a 25-gauge needle. This needle was previously rinsed with marine anticoagulant (0.45 M NaCI; 0.1 M glucose; 30 mM trisodium citrate; 26 mM citric acid, and 10 mM EDTA; pH 4.6) [36] and samples were subsequently fixed in molecular-grade ethanol.

### Tissue histology

Sections of gonads, heart, hepatopancreas, gills and muscle were placed into histological cassettes and submerged in Davidson’s Saltwater Fixative (DSF) [37]. After a 24-h period, samples were transferred to 70% industrial methylated spirit and sent to Cefas Weymouth Laboratory (UK) for further processing and analysis. Fixed samples were processed to wax using a vacuum infiltration processor following standard protocols [35, 38]. Sections were cut at a thickness of 3 to 5 μm on a rotary microtome and then mounted onto glass slides before staining with haematoxylin and eosin (HE). Stained sections were analysed using light microscopy (Nikon Eclipse E800) and digital images were captured using the Lucia^™^ Screen Measurement System (Nikon) and SLIDEVIEW^™^ VS200 Research Slide Scanner (Olympus Life Science). Tissue histology was conducted on n = 122 samples, serving as an additional tool to PCR for screening the presence of *Hematodinium* sp. in tissues from the ‘pathobiome-set’. The presence of *Hematodinium* sp. infection in tissues was evaluated using a simple scoring system (0 and 1). In this system, a score of 0 indicates no visible presence of *Hematodinium* sp., whereas a score of 1 denotes the presence of parasites in the tissue.

### DNA extraction

DNA from the haemolymph (n = 195) and gut (n = 200) of the ‘pathobiome-set’ was extracted using the Promega Maxwell® RSC Tissue DNA Kit (Promega, United Kingdom) following the manufacturer's protocol. Prior to extraction, ethanol was removed from the samples by freeze-drying. Dried samples were resuspended in 700 µL of Lifton's buffer for haemolymph and 600 µl for gut samples, and then shaken using a bead beater for 2 min at a speed of 500 rpm. Subsequently, samples were centrifuged for 2 min at 9,000 rpm, and 20 µl of Proteinase K (10 ug/mL) was added. The samples were digested overnight at 57 °C before extraction. DNA was then eluted in 100 µl of elution buffer. The purity and concentration of the extracted DNA were evaluated using a NanoDrop ND-1000 Spectrophotometer (Thermo Fisher Scientific, United Kingdom). Concentrations were further confirmed using a Qubit 2.0 Fluorometer (Thermo Fisher Scientific, United Kingdom).

### Molecular detection of ***Hematodinium*** sp.

The presence of *Hematodinium* sp. in haemolymph samples was performed using PCR amplification of the ITS1 region. To design specific primers, we utilized the representative sequence DQ871209 of *Hematodinium* sp. from *N. norvegicus* as a template in Primer3 software [39]. The forward primer HSPP-F1 (5′-GGAAGTTTAGTGAACCTTATCAC-3′, Tm = 68.2 °C) and the reverse primer HSPP-R1 (5′-CAGCCTTTTCCAGTTTCTGGAAG-3′, Tm = 71.8 °C) were designed for the amplification of *Hematodinium* sp. PCR was performed in a total reaction volume of 25 µl, using GoTaq® Green Master Mix (Promega, UK). Positive and negative controls were run alongside all screening PCR reactions. The PCR was conducted on a Systemics^™^ PCR machine using the following thermal profile: initial denaturation at 94 °C for 5 min, then 35 cycles of denaturation at 94 °C for 30 s, annealing at 56 °C for 30 s and extension at 72 °C for 1 min 30 s, with a final extension of 72 °C for 5 min. A 5 µL aliquot of each PCR product was checked using 1.5% agarose gel containing 0.1 μg/mL ethidium bromide in Tris–Acetate-EDTA (TAE) buffer. Images were captured with a BioRad^™^ gel documentation system. For sequence verification, the PCR amplicons were purified using a WIZARD clean-up kit, in accordance with the manufacturer's instructions, and subsequently sent for sequencing to Eurofins Genomics, UK.

### Bacterial 16S rRNA amplicon sequencing

The extracted DNA was sent to Novogene (Cambridge, United Kingdom) for 16S rRNA amplicon library preparation and sequencing. Out of the original n = 195 haemolymph samples, n = 122 (representing 63%) passed Novogene’s quality control checks (DNA purity and quantity) and were therefore processed for library preparation and sequencing, along with the corresponding gut samples (n = 122). The V4 hypervariable region of the bacterial 16S rRNA gene (~ 292 bp) was amplified using the primers 515 F (GTGCCAGCMGCCGCGGTAA) and 806R (GGACTACHVGGGTWTCTAAT). Sequencing was carried out by Novogene on an Illumina paired-end platform (Illumina PE250 platform) using an S4 flowcell on an Illumina Novaseq (Illumina, United States). Sequencing quality control metrics and data treatment procedures are shown in Supplementary Table S1. Data rarefaction was not performed in this study to avoid the potential loss of valuable information and bias in abundance estimation [40].

### Bioinformatics and statistical analysis

Data processing was conducted on an HP workstation with 32 processors using Debian Linux (version 10). A custom Python pipeline was created to employ Mothur's SOP [41] and the SILVA reference database [42] for processing raw PE (fastq files) involving sequence cleaning, operational taxonomic units (OTUs) clustering, and taxonomical classifications [43]. To enhance the efficiency of high-throughput analysis of numerous sample sets, the pipeline was segmented into distinct tasks. Each task was executed sequentially by running Mothur in batch mode. Python scripts that generated the required Mothur commands for each sample fastq and the associated mock fastqs were used. Both haemolymph and gut data were processed through Mothur to compare them, and then each was run separately for further comparisons. Statistical analyses were conducted in R Studio (Version R-4.1.2), using the Phyloseq package to compute alpha diversity [44]. The homogeneity of alpha-diversity indices' variance was validated using the Shapiro–Wilk test before assessing the differences between groups. If data were normally distributed, alpha diversity indices were analysed using one-way ANOVA, with further pairwise comparisons made using a t-test. Alternatively, for non-normally distributed data, the Kruskal–Wallis test was applied, and further pairwise comparisons used the Wilcoxon test (rank-sum test). The Benjamini and Hochberg (BH) correction [45] was utilised to adjust *p*-values, with statistical analysis conducted using the rstatix package [46]. All figures were generated using the R packages ggpubr and ggplot2 [46, 47]. Beta-diversity comparisons were computed using the Bray–Curtis pairwise distances with vegan [48] and Phyloseq packages, and visualized using Principal Coordinate Analysis (PCoA). Group differences were calculated using the Vegan package's non-parametric permutational multivariate ANOVA (PERMANOVA) with 1,002 permutations. Beta diversity dispersion was assessed using PERMDISP (permutational analysis of multivariate dispersions) from the vegan package with Bray–Curtis dissimilarity. Group dispersion differences were tested to evaluate heterogeneity in community structure. An adjusted *p*-value less than 0.05 was deemed statistically significant. The shared taxa and core microbiome and shared communities were identified using the ampvis2 package [49]. Both the observed and abundant OTUs in both tissues were defined as the core communities. The average abundance of each OTU in all the samples from a given tissue was computed, divided by the total abundance of all OTUs in that tissue. The cumulative OTU read abundance for each tissue was calculated. The Wilcoxon rank-sum test was used for comparisons of the same taxa in different groups, followed by BH false discovery rate (FDR) correction with a 0.05 *p*-value.

## Results

### Prevalence of ***Hematodinium*** sp. in ***Nephrops norvegicus*** from the Clyde Sea area (CSA): The'prevalence-set'

The body colour method (BCM) and pleopod method (PM) were used to evaluate the levels of patent *Hematodinium* sp. infection in *N. norvegicus* collected from the CSA (study area) during the period of this research. Based on the BCM, 17% of *N. norvegicus* showed a high-intensity level of infection (BCM positive), while around 26% were shown to be infected according to the PM (compiled stages S1–S4). No significant differences in patent infection were found between the sexes. Detailed data collected from the'prevalence-set'can be found in Table 1.

**Table 1:** Summary data for the ‘prevalence-set’ of *N. norvegicus* collected from the Clyde Sea Area

Parameters	Female		Male		Total	
	**N**	**%**	**N**	**%**	**N**	**%**
Sex	46	30.87	103	69.13	149	100
Body colour method (BCM)						
Negative (BCM)	37	29.84	87	70.16	124	83.22
Positive (BCM)	9	36	16	64	25	16.78
Pleopod method (PM)						
S0	34	30.63	77	69.37	111	74.50
S1	4	21.05	15	78.95	19	12.75
S2	3	37.50	5	62.50	8	5.37
S3	5	50	5	50	10	6.71
S4	1	100	0	0	1	0.67
S1–4	12	31.58	26	68.42	38	25.50
Moulting parameters						
Hard (H)	39	28.89	96	71.11	135	90.60
Soft (S)	7	50	7	50.00	14	9.40
RM (recently moulted)	5	45.45	6	54.55	11	7.38
IM (inter-moult)	28	27.72	73	72.28	101	67.79
A + B	5	45.45	6	54.55	11	7.38
C	28	27.72	73	72.28	101	67.79
D	13	35.14	24	64.86	37	24.83
Size parameter						
Immature	10	20.41	39	79.59	49	32.89
Mature	36	36	64	64.00	100	67.11
S (23–25 mm)	5	62.50	3	37.50	8	5.37
M (25–30 mm)	33	43.42	43	56.58	76	51.01
L (30–49.7 mm)	8	12.31	57	87.69	65	43.62

###  Infection profiles of the ‘pathobiome-set’ samples

To investigate the potential existence of a pathobiome associated with *Hematodinium* sp. infection, a total of 200 *N. norvegicus* were selected from the catch. The selection was based on their visible characteristics, including both those with and without visible signs of disease (BCM positive and negative). These selected specimens were processed and both data and samples were collected for subsequent analysis on the same day. However, following the DNA extraction from the haemolymph, a total of n = 122 samples met the quality control standards and were thus fully analysed. Out of this subset (n = 122), 44.3% were BCM positive (n = 54) and 55.7% were BCM negative (n = 68). Of the BCM negative specimens, a total of n = 66 were also confirmed negative according to the PM. However, after the histological and molecular screening, a total of n = 45 (out of n = 66) were confirmed as ‘*Hematodinium*-free’ (negative by both histology and PCR). The histological analysis revealed a strong correlation between the observed level of infection in the tissue sections and the pleopod scoring. The gonads, the heart and the hepatopancreas were the most frequently colonised tissues, followed by gills and muscle (Supplementary Figs. 1, 2, 3, 4, 5 and 6). In heavily infected *N. norvegicus*, *Hematodinium* cells occluded the haemocoel spaces, causing the rupture of the tissue. No signs of cell-directed response were observed against *Hematodinium* and no co-infections were visually apparent either. *Hematodinium* trophonts presented a variety of oval shapes and could have one or more nuclei. Information on the number of samples available for different groupings based on the different methods used to determine *Hematodinium* sp. infection severity is depicted in Table 2.

**Table 2:** Summary data for the ‘pathobiome-set’ of *N. norvegicus* collected from the Clyde Sea Area

Parameters	Female		Male		Total	
	**N**	**%**	**N**	**%**	**N**	**%**
Sex	56	45.9	66	54.1	122	100
Body colour method (BCM)						
Negative (BCM)	29	42.65	39	57.35	68	55.74
Positive (BCM)	27	50	27	50	54	44.26
Pleopod method (PM)						
S0	28	42.4	38	57.6	66	54.1
S1	5	41.7	7	58.3	12	9.8
S2	11	52.4	10	47.6	21	17.2
S3	4	36.4	7	63.6	11	9.0
S4	8	66.7	4	33.3	12	9.8
S1–4	28	50	28	50	56	45.9
Molecular diagnostic (PCR)						
Negative (PCR)	20	35.7	28	42.4	48	39.3
Positive (PCR)	36	64.3	38	57.6	74	60.7
Histology						
Negative (Histology)	19	38.8	30	61.2	49	40.16
Positive (Histology)	37	50.7	36	49.3	73	59.84
Negative (gill)	22	40.74	32	59.26	54	44.26
Positive (gill)	34	50	34	50.00	68	55.74
Negative (gonad)	24	54.55	20	45.45	44	36.07
Positive (gonad)	27	71.05	11	28.95	38	31.15
Negative (heart)	22	40.74	32	59.26	54	44.26
Positive (heart)	30	50.85	29	49.15	59	48.36
Negative (hepatopancreas)	23	41.82	32	58.18	55	45.08
Positive (hepatopancreas)	33	49.25	34	50.75	67	54.92
Negative (muscle)	23	44.23	29	55.77	52	42.62
Positive (muscle)	33	47.14	37	52.86	70	57.38
Histology/PCR						
Histology/PCR (Negative/Negative)	18	40	27	60	45	36.9
Histology/PCR (Positive/Negative)	2	66.7	1	33.3	3	2.5
Histology/PCR (Negative/Positive)	1	25	3	75	4	3.3
Histology/PCR (Positive/Positive)	35	50	35	50	70	57.4
Moulting stage						
Hard (H)	45	42.86	60	57.14	105	86.07
Soft (S)	11	64.71	6	35.29	17	13.93
RM (recently-moulted)	17	54.84	14	45.16	31	25.41
IM (inter-moult)	39	42.86	52	57.14	91	74.59
B	11	68.75	5	31.25	16	13.11
C	39	43.33	51	56.67	90	73.77
D	6	37.5	10	62.50	16	13.11
Size range						
Immature	13	25	39	75	52	42.62
Mature	43	61.43	27	38.57	70	57.38
S (22.6–25 mm)	8	88.89	1	11.11	9	7.38
M (25–30 mm)	42	46.15	49	53.85	91	74.59
L (30–38.90 mm)	6	27.27	16	72.73	22	18.03

### Composition of the haemolymph and gut microbiomes in ‘healthy’ *Nephrops norvegicus* (*Hematodinium*-free)

**Fig. 1:**
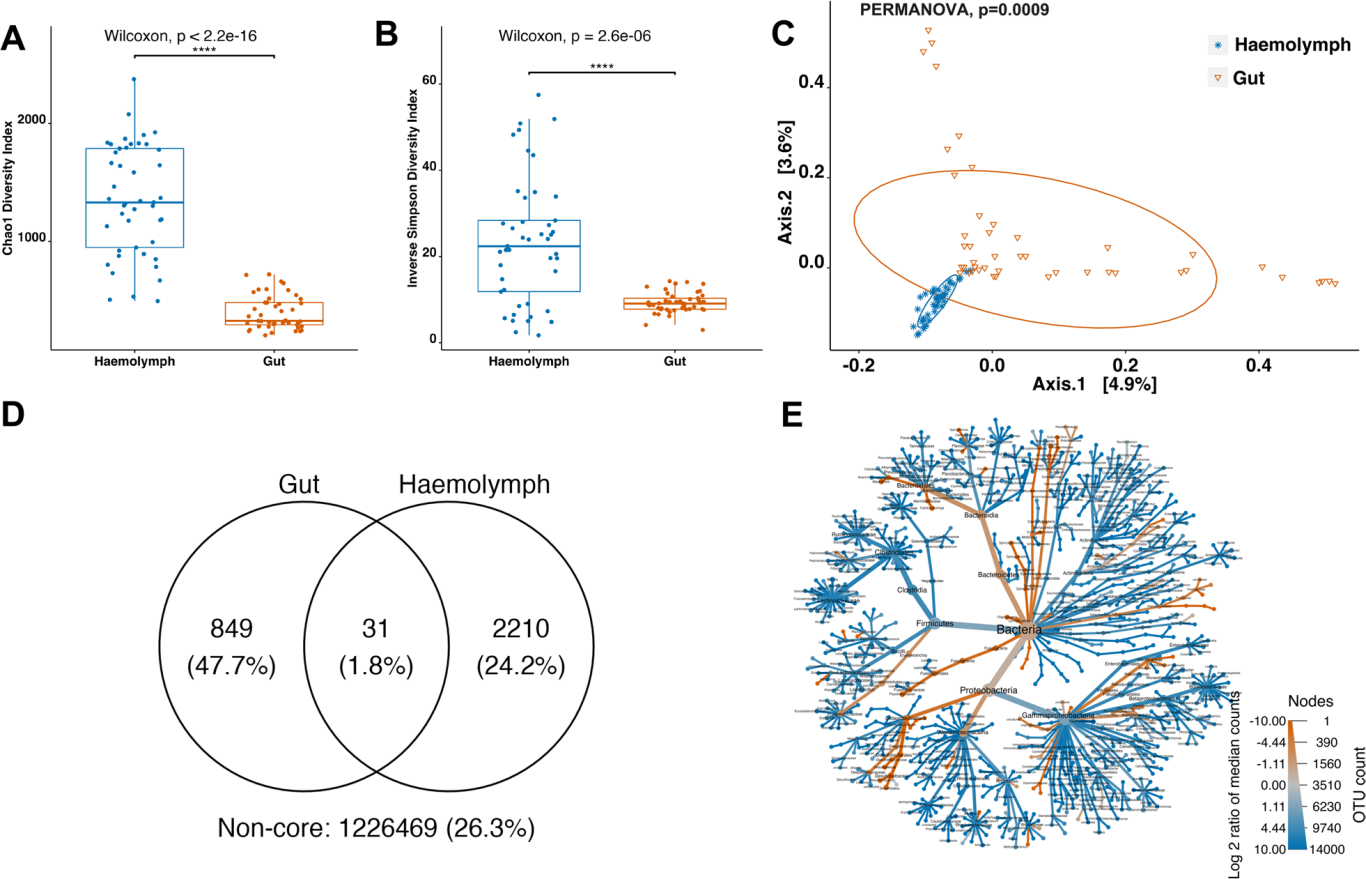
Differential microbial community composition of the haemolymph and gut microbiomes in ‘healthy’ *N. norvegicus* (n = 45). Alpha-diversity indices, **A** Chao1 and **B** Inverse Simpson, were significantly higher in haemolymph (Wilcoxon, *p* < 0.0001). Each dot represents a sample, and **** indicates *p* < 0.0001. **C** Principal Coordinate Analysis (PCoA) plot based on Bray–Curtis dissimilarity matrix of haemolymph and gut communities (PERMANOVA, *p* = 0.0009). The colours of ellipses and the shape of individual samples represent the two groups. **D** Venn diagram of shared and unique operational taxonomic units (OTUs) between haemolymph and gut. The numbers of OTUs with at least 0.1% relative abundance are displayed. Numbers in brackets represent the average relative abundance of the OTUs in that group. **E** Metacoder heat-tree showing taxonomic differences between haemolymph and gut. Nodes correspond to phylotypes, while edges link phylotypes following the taxonomic hierarchy. Node sizes correspond to the number of observed OTUs. Colours represent the log fold difference of a given phylotype's median relative abundance in haemolymph compared to the gut. Taxa-coloured blue indicate significant enrichment in the haemolymph, while taxa-coloured red are enriched in the gut

The haemolymph and gut microbiomes of n = 45 ‘healthy’ *N. norvegicus* (*Hematodinium*-negative by BCM, PM, histology tissue screening and haemolymph-PCR) were examined to characterise the structure of microbial communities and identify their core microbiome and shared taxa. Alpha-diversity indices, Chao1 and Inverse Simpson, were computed to identify the overall community richness, diversity and evenness between haemolymph and gut. The haemolymph microbial community indicated higher bacterial richness (Chao1, Wilcoxon, *p* < 0.00001; Fig. 1A) and evenness (Inverse Simpson, Wilcoxon, *p* < 0.00001; Fig. 1B) compared to the gut. Beta diversity analysis based on Bray–Curtis dissimilarity indicated significant differences in the overall microbial community composition between haemolymph and gut (PERMANOVA, *p* = 0.0009). The data were illustrated in the PCoA matrix as shown in Fig. 1C. PERMDISP indicated no significant difference in dispersion between the two groups (*p* = 0.5165), confirming that the observed community differences were not driven by heterogeneity in variance. A total of 31 shared OTUs between haemolymph and gut were identified (Fig. 1D), belonging to the genera *Enterobacteriaceae unclassified, Pseudomonas, Muribaculaceae ge, Streptococcus, Ralstonia, Muribaculaceae unclassified, Bacillaceae unclassified, Subdoligranulum, Enterococcaceae unclassified, Helicobacter, and Erysipelatoclostridium* (Supplementary Table S2). The heat tree illustrated in Fig. 1E highlights the significantly different abundant taxa between haemolymph and gut for taxa with a relative abundance > 0.1%.

**Fig. 2:**
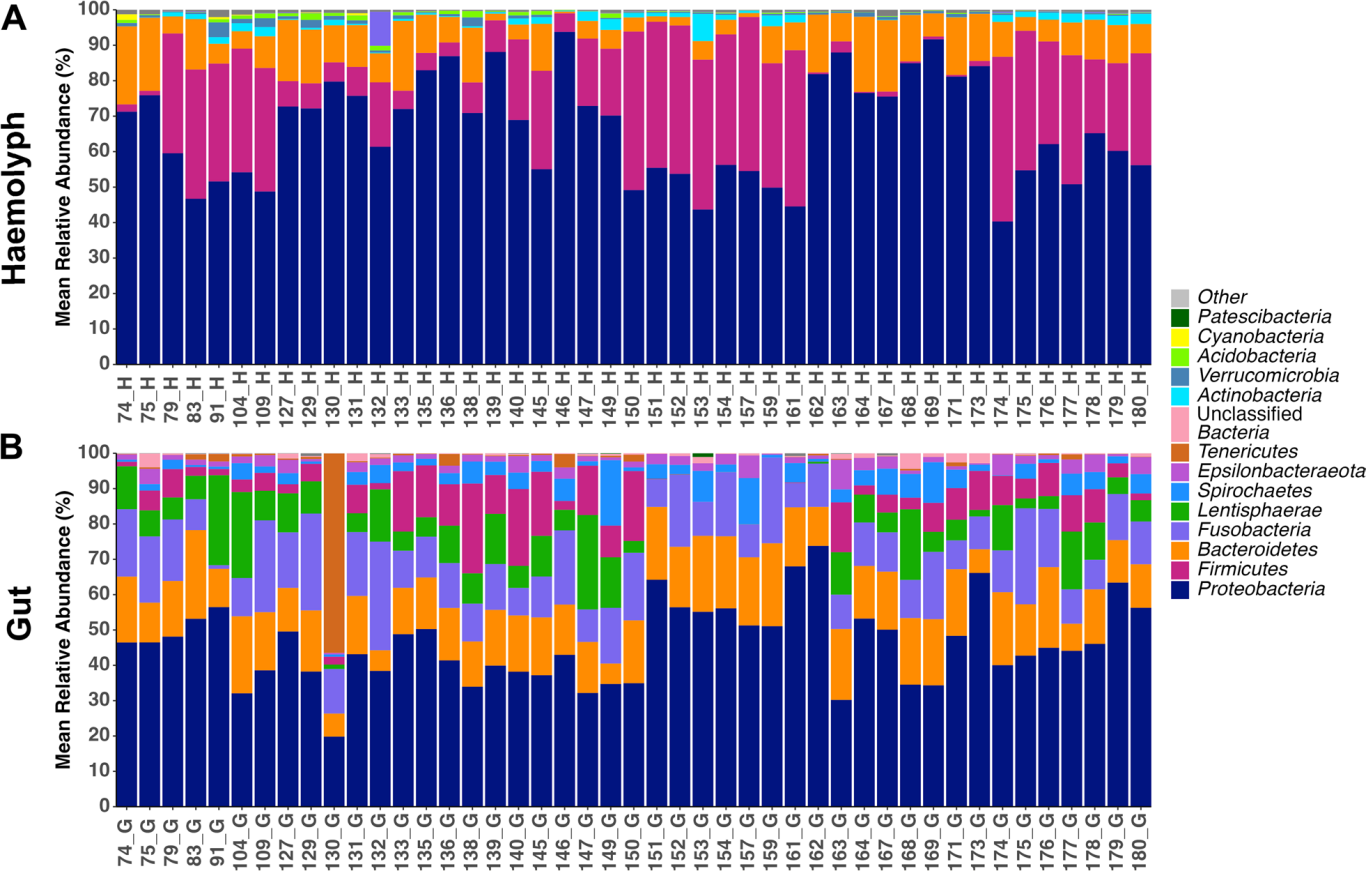
Mean relative abundance (%) of the microbial composition at phylum level observed in the **A** haemolymph and **B** gut of ‘healthy’ *N. norvegicus, ‘Hematodinium*-negative’ individuals confirmed by several methods: BCM, PM, PCR and histology (n = 45). Phyla contributing more than 1% to the overall abundance are individually reported, while those with an abundance of less than 1% are collectively categorised as “Other”

The most dominant phyla in the haemolymph were *Proteobacteria* (66.48 ± 14.66%), *Firmicutes* (20.29 ± 16.37%), *Bacteroidetes* (9.72 ± 5.87%), and *Actinobacteria* (1.28 ± 1.38%) (Fig. 2A). In the gut, the dominant phyla were *Proteobacteria* (46.11 ± 11.12%), *Bacteroidetes* (15.66 ± 4.69%), *Fusobacteria* (14.15 ± 6.26%), *Lentisphaerae* (8.00 ± 6.85), *Firmicutes* (7.00 ± 6.47%), *Spirochaetes* (3.99 ± 3.58%), *Epsilonbacteraeota* (2.46 ± 1.73%), and *Tenericutes* (1.76 ± 8.37%) (Fig. 2B). The families *Enterobacteriaceae* (35.66 ± 15.99%) and *Lachnospiraceae* (9.16 ± 8.38) were most abundant in the haemolymph, while *Unclassified Alphaproteobacteria* (14.74 ± 8.30%) and *Fusobacteriaceae* (14.15 ± 6.26%) were the most abundant in the gut. The core haemolymph microbiome, representing 32.6% of total reads, consisted of 115 genera belonging to 68 families within 7 phyla (*Acidobacteria*, *Actinobacteria*, *Bacteroidetes*, *Epsilonbacteraeota*, *Firmicutes*, *Proteobacteria,* and *Verrucomicrobia*) (Supplementary Table S3). On the other hand, the gut core microbiome, comprising 74.7% of the total reads, included 33 genera within 27 families spanning 9 phyla (*Bacteroidetes, Epsilonbacteraeota, Firmicutes, Fusobacteria, Lentisphaerae, Proteobacteria, Spirochaetes, Tenericutes,* and *Bacteria unclassified*) (Supplementary Table S4).

### Comparative analysis of haemolymph and gut microbiomes in small and large ‘healthy’ *Nephrops norvegicus* (*Hematodinium*-free)

**Fig. 3:**
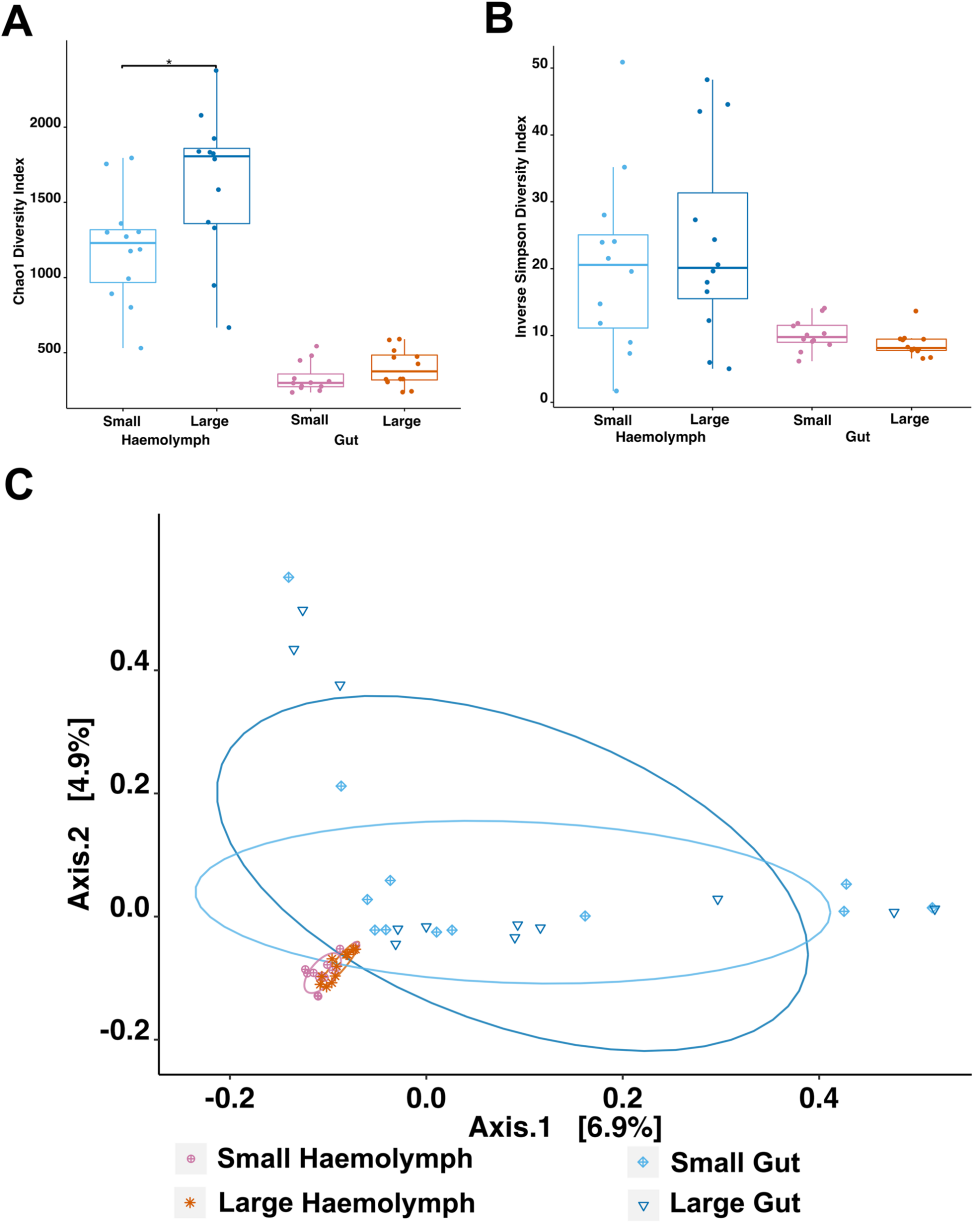
Differential microbial community composition of the haemolymph and gut microbiomes in small and large ‘healthy’ *N. norvegicus* (n = 12/group). Alpha-diversity indices, **A** Chao1 and **B** Inverse Simpson, of small and large *N. norvegicus* haemolymph and gut microbial communities are presented. Each dot represents a sample and * indicates *p* < 0.05. **C** Principal Coordinate Analysis (PCoA) plot based on Bray–Curtis dissimilarity matrix of haemolymph and gut communities of small and large *N. norvegicus*. The colours of ellipses and the shape of individual samples represent the groups

In our recent study [17], we observed that smaller animals are more likely to develop a patent infection. We sought to explore if smaller animals have differential microbiomes that could make them more susceptible to disease progression. Here, we compared haemolymph and gut microbiomes of small (n = 12, 25.2 ± 1.20 mm CL) and large (n = 12, 31.9 ± 1.71 mm CL) ‘healthy’ *N. norvegicus (Hematodinium*-negative by all methods). Our data highlighted that the haemolymph microbiome of large animals showed higher richness (Chao1, Wilcoxon, *p* = 0.012; Fig. 3A). However, no significant differences were observed in pairwise comparisons of the haemolymph and gut microbiomes between large and small animals for community evenness and beta diversity (Figs. 3B, C), and PERMDISP analysis indicated no significant variation in dispersion across the four groups (*p* = 0.7567).

### Pathobiome associated with subpatent levels of ***Hematodinium*** sp.

**Fig. 4:**
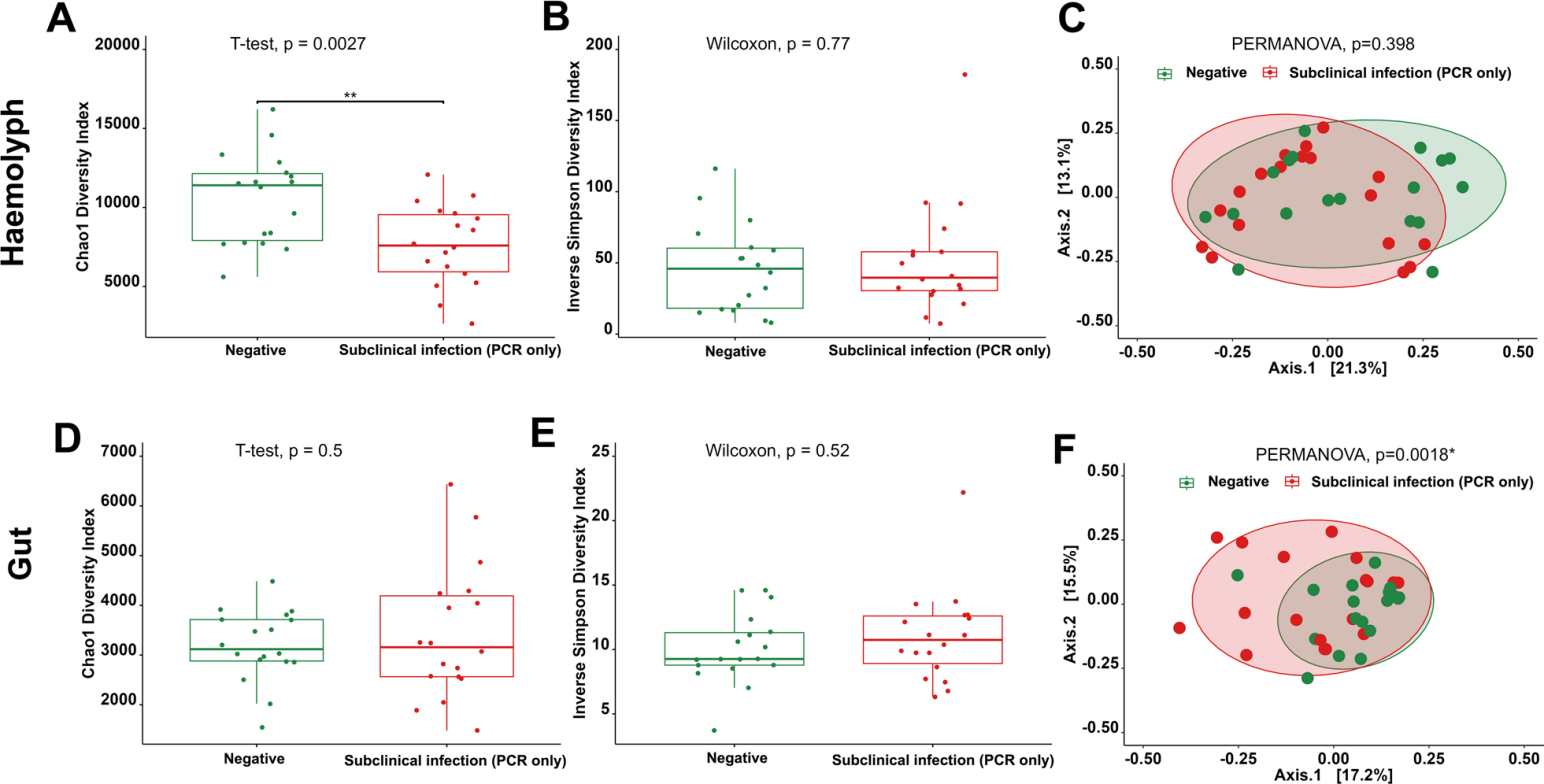
Comparison of haemolymph and gut microbial communities of '*Hematodinium-*free' *N. norvegicus* (confirmed negative by BCM, PM, PCR, histology; n = 18) and subpatent-infected animals (confirmed positive by PCR and histology but negative according to BCM and PM; n = 18). Alpha-diversity metrics for the haemolymph (**A**, **B**) and gut (**D**, **E**) comparisons are depicted. Principal coordinate analysis (PCoA) plots based on Bray–Curtis similarity matrix are shown for the haemolymph (**C**) and gut (**F**) microbial communities. The colours of the ellipses represent the two experimental groups

Initially, our objective was to assess any differences in microbial assemblages associated with the early stages of *Hematodinium* sp. infection compared to uninfected individuals. To this end, we compared '*Hematodinium*-free' samples with samples that tested positive with only PCR and histology but were categorised negative according to the PM and BCM (n = 18). In this case, the haemolymph of subpatent *Hematodinium* sp. positive individuals showed a significant reduction in bacterial species richness (Chao1, t-test, *p* = 0.0027; Fig. 4A), although no significant differences were noted in terms of diversity or evenness (Fig. 4B) and beta diversity (Fig. 4C). PERMDISP indicated no significant variation in dispersion between the haemolymph of positive and negative groups (*p* = 0.3051). In contrast, no differences in gut bacterial richness, diversity and evenness were observed (Fig. 4D, E). However, as illustrated in Fig. 6F, the clustering of ‘*Hematodinium*-free’ samples from the gut was more evident, and the PERMANOVA results displayed significant divergence (PERMANOVA, *p* = 0.0018). PERMDISP analysis for gut samples showed no significant differences in dispersion between positive and negative groups (*p* = 0.07278).

### Pathobiome associated with advanced stages of *Hematodinium* sp. infection

**Fig. 5:**
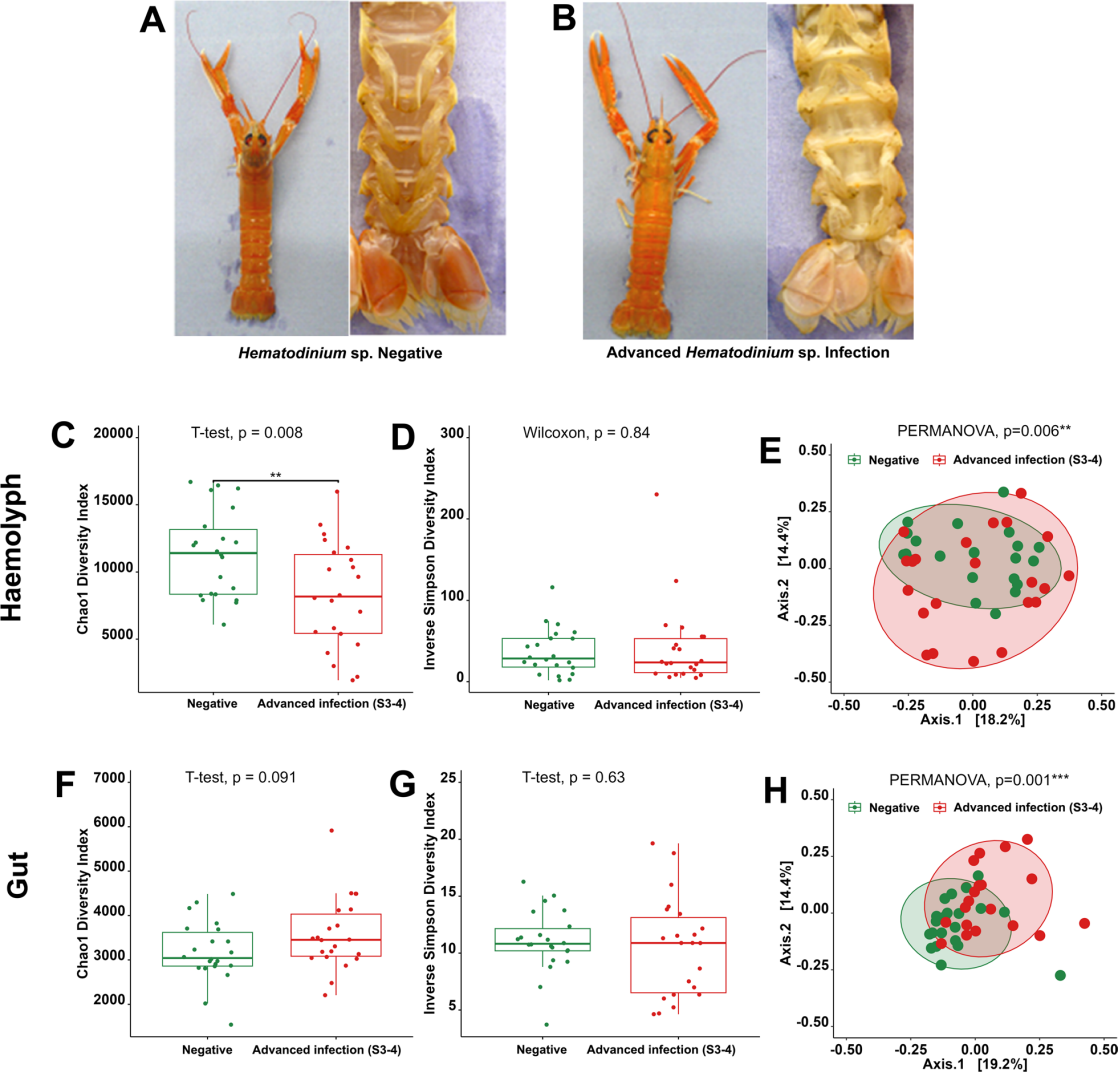
Differences in *N. norvegicus* between **A** '*Hematodinium-* free' and **B** advanced *Hematodinium* sp. infection, categorised using BCM. Heavily infected *N*. *norvegicus* display abnormal yellow/orange body colouration, discolouration or pigment loss within extremities, opacity of the musculature between the limb joints and a milky-white appearance of the haemolymph. Sections (**C–H)** illustrate a comparison of haemolymph and gut microbial communities between ‘*Hematodinium*-free’ *N.  norvegicus* (tested negative by BCM, PM, PCR, histology; n = 22) and animals at advanced levels of infection (confirmed positive by BCM and PM stages 3–4; n = 22). Alpha-diversity metrics of the haemolymph (**C**, **D**) and gut (**F**, **G**) comparisons are shown. Each dot represents an individual sample, and **p < 0.001. Principal Coordinate Analysis (PCoA) plots based on Bray–Curtis similarity matrix are depicted for the haemolymph (**E**) and gut (**H**) microbial communities. The colours of the ellipses correspond to the two groups

**Fig. 6:**
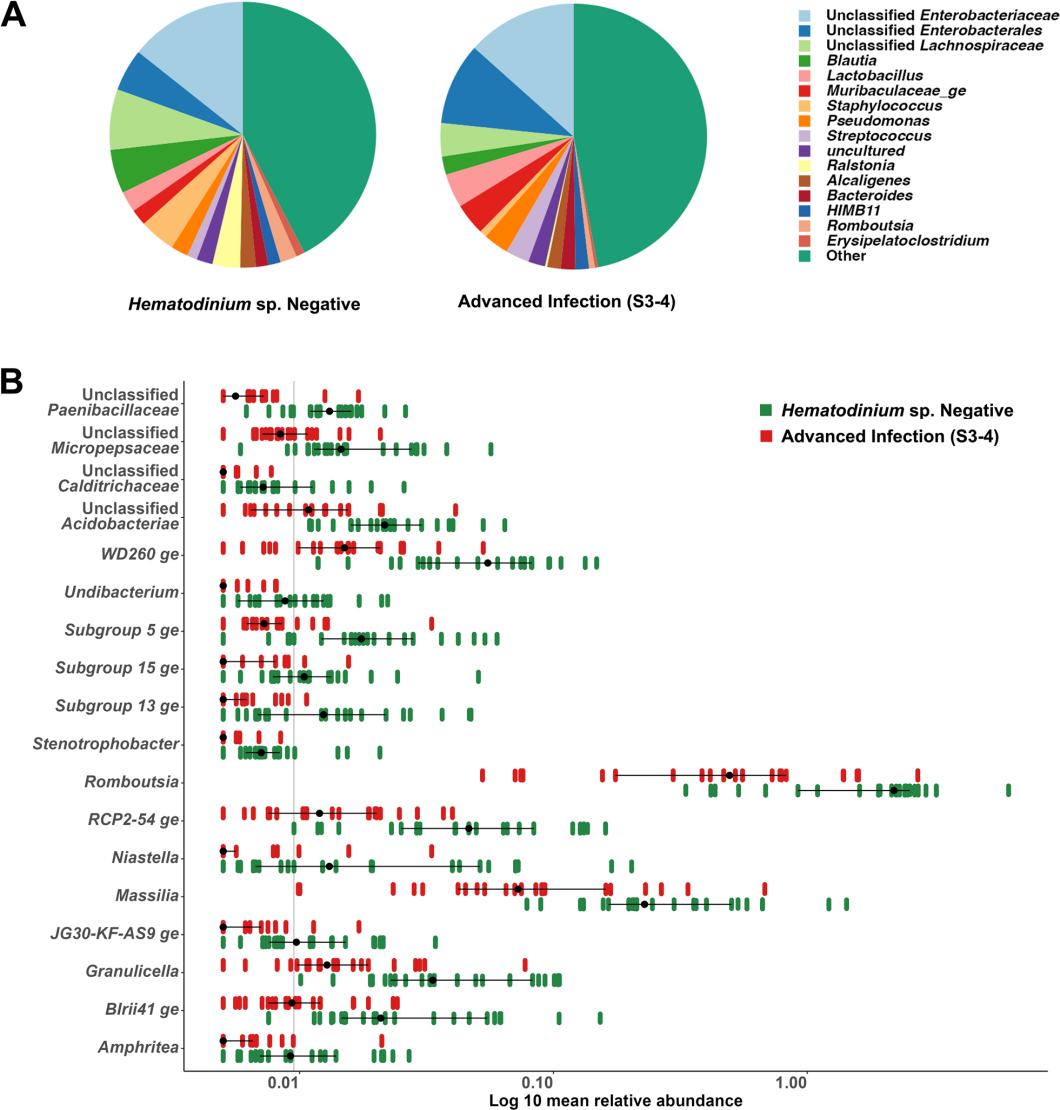
Differential abundance of genera in the haemolymph of *'Hematodinium-*free' *N. norvegicus* (tested negative by BCM, PM, PCR, histology; n = 22) compared to individuals with advanced levels of infection (confirmed positive by BCM and PM stages 3–4; n = 22). **A** A pie chart illustrates the mean relative abundance (%) of the most prevalent genera in the haemolymph of ‘*Hematodinium*-free’ *N. norvegicus* and those with advanced infection. Genera contributing more than 1% to the overall abundance are individually reported, while those with an abundance of less than 1% are collectively categorised as “Other”. **B** Significantly different genera (*p* < 0.01) were observed in the haemolymph microbiome between *'Hematodinium*-free' *N. norvegicus* and those with advanced levels of infection

**Fig. 7:**
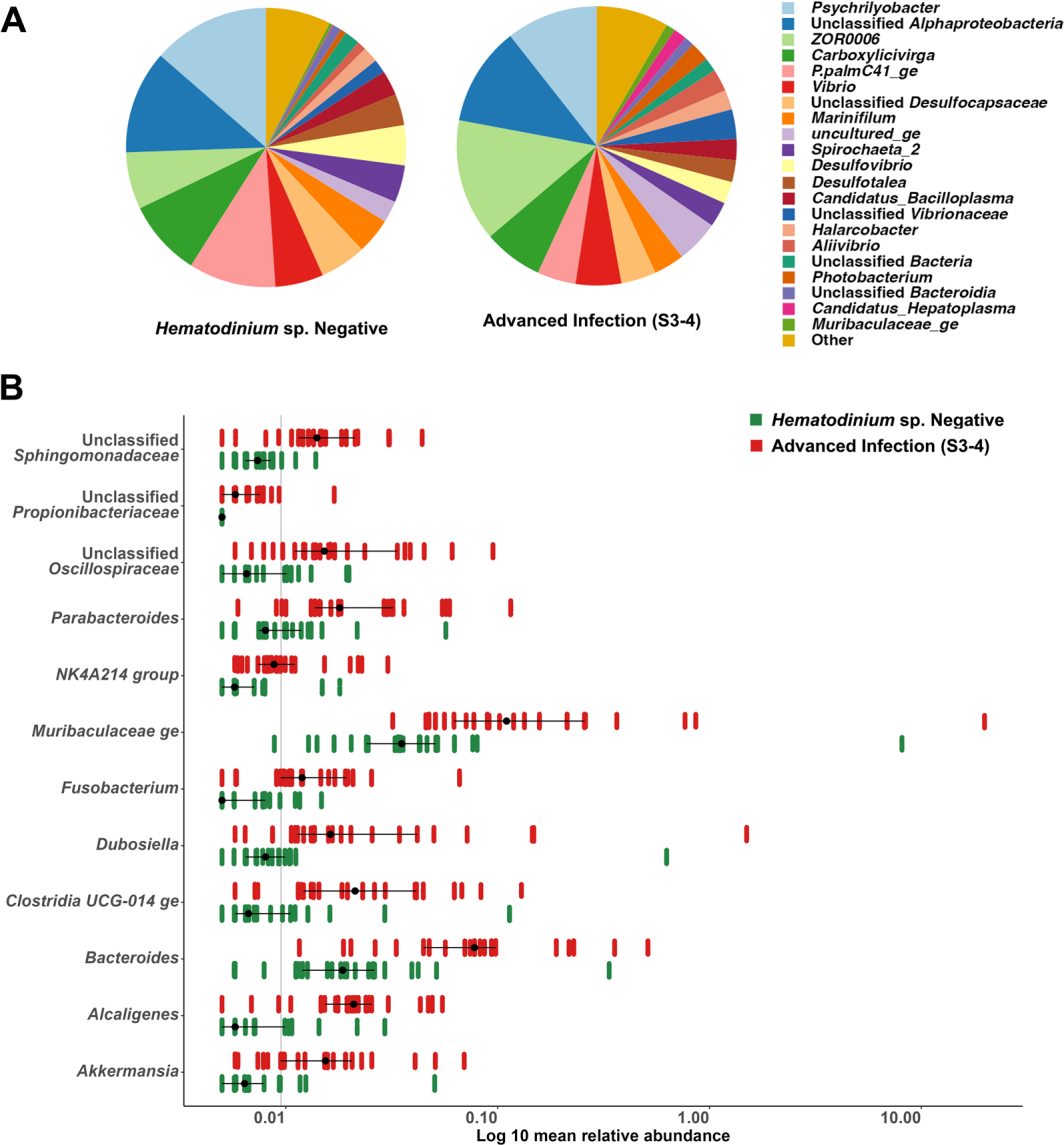
Differential abundance of genera in the gut of *'Hematodinium-*free' *N.  norvegicus* (tested negative by BCM, PM, PCR, histology; n = 22) compared to individuals with advanced levels of infection (confirmed positive by BCM and PM stages 3–4; n = 22). **A** A pie chart illustrates the mean relative abundance (%) of the most prevalent genera in the haemolymph of ‘*Hematodinium*-free’ *N. norvegicus* and those with advanced infection. Genera contributing more than 1% to the overall abundance are individually reported, while those with an abundance of less than 1% are collectively categorised as “Other”. **B** Significantly different genera (*p* < 0.01) were observed in the gut microbiome between *'Hematodinium*-free' *N. norvegicus* and those with advanced levels of infection

To evaluate the impact of advanced infection, we compared haemolymph and gut microbiome data from '*Hematodinium*-free' *N. norvegicus* (negative by all methods), shown in Fig. 5A, to data from BCM and PM stages 3–4 positive*N. norvegicus*, depicted in Fig. 5B (n = 22). The bacterial community richness in haemolymph, as indicated by the Chao1 index, was significantly reduced in *N. norvegicus* in advanced stages of infection (Chao1, t-test, *p* = 0.008; Fig. 5C). However, no significant differences were found regarding diversity and evenness (Inverse Simpson index, Wilcoxon, *p* = 0.84; Fig. 5D). Significant dissimilarities between '*Hematodinium*-free' and *N. norvegicus* at advanced stages of infection were identified (PERMANOVA, *p* = 0.006; Fig. 5E). PERMDISP analysis highlighted no significant differences between groups (*p* = 0.0594). In contrast, in the gut, there were no significant differences in microbial richness (Fig. 5F) or diversity and evenness (Fig. 5G) between '*Hematodinium*-free' and *N. norvegicus* at advanced stages of infection. Nevertheless, the '*Hematodinium*-free' samples exhibited a tighter clustering in the PCoA (axis 1), and the PERMANOVA analysis revealed significant differences between the two groups (PERMANOVA, *p* = 0.001; Fig. 5H). PERMDISP analysis indicated no significant differences (*p* = 0.0693). Furthermore, we identified differentially abundant bacterial genera between '*Hematodinium*-free' *N. norvegicus* and those with advanced levels of infection (Figs. 6, 7). In total, 145 genera in the haemolymph and 88 in the gut exhibited significant differences (Wilcoxon,* p* < 0.05; Supplementary Tables S5-6). Among these, 18 genera in the haemolymph and 12 in the gut displayed highly significant differences (Wilcoxon, *p* < 0.01; Figs. 6B, 7B). In the haemolymph, typically differentially abundant genera were characterised by a significant decrease in *N.* *norvegicus *at advanced stages of infection (e.g., *Romboutsia*, *Niastella* and *Amphritea*). In contrast, in the gut, the observed differences were marked by a significant increase in certain genera, such as *Muribaculaceae*, *Fusobacterium* and *Dubosiella*, in *N. norvegicus* at advanced stages of infection.

### Microbiome dynamics associated with *Hematodinium* sp. disease progression

**Fig. 8:**
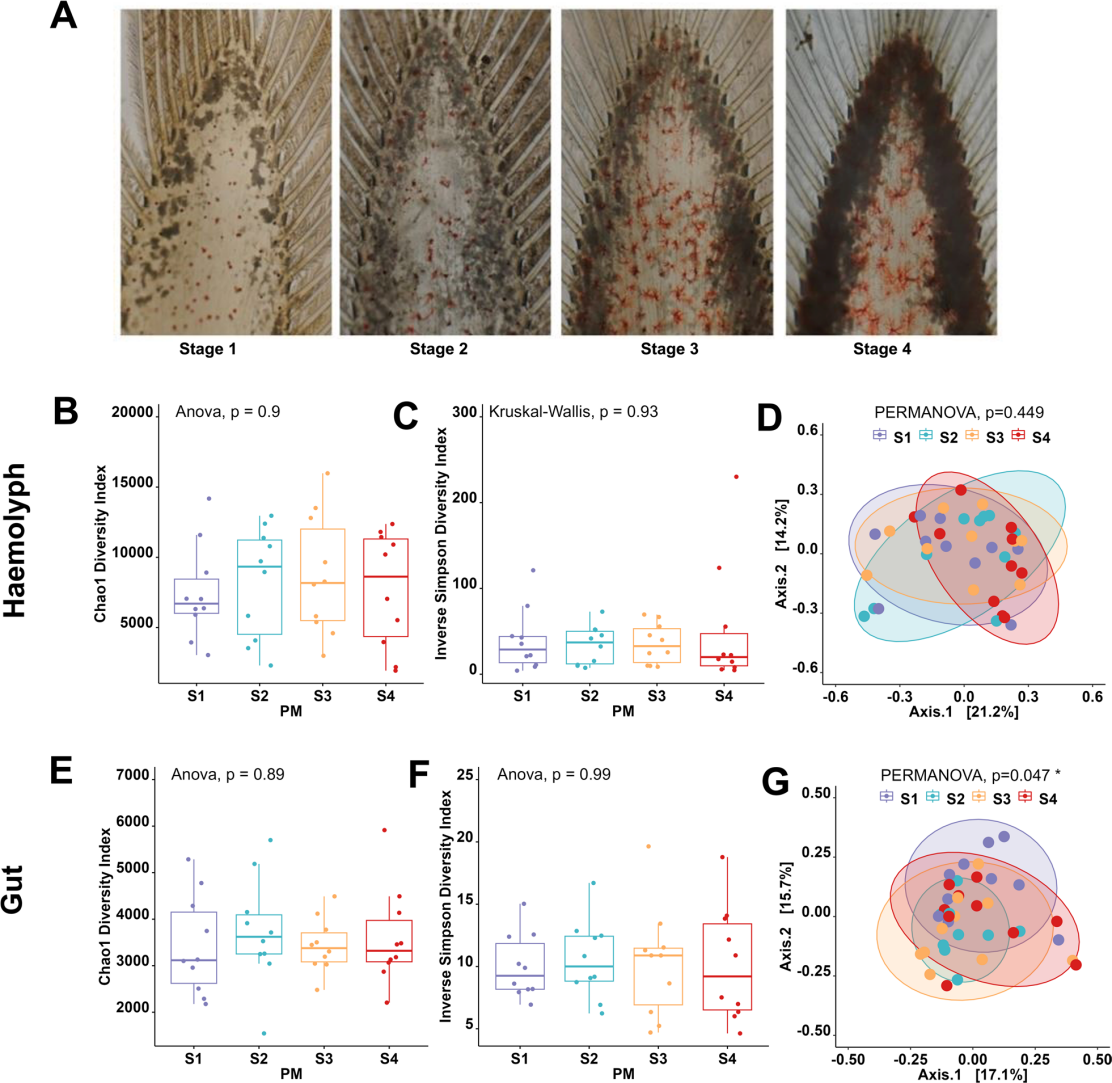
Comparison of haemolymph and gut microbial communities of *N. norvegicus* through the progression of *Hematodinium* sp. infection, based on the pleopod method scoring system (stages 1–4; n = 10). **A** Pleopod scoring for *Hematodinium* sp. infection in *N. norvegicus* is used to identify dense aggregations of the parasite, which appear as darkened areas; these can be scored on a scale from 1 to 4, reflecting the intensity of infection. Alpha-diversity metrics for the haemolymph (**B**, **C**) and gut (**E**, **F**) comparisons are depicted. Principal Coordinate Analysis (PCoA) plots based on Bray–Curtis similarity matrix are shown for the haemolymph (**D**) and gut (**G**) microbial communities. The colours of the ellipses correspond to the four stages of infection

To investigate the dynamics of the microbiome in relation to *Hematodinium* sp. disease progression, we compared samples from various stages of infection (stages 1–4; n = 10). as classified by PM (Fig. 8A). As indicated in Fig. 8, no significant differences in bacterial community richness, diversity, or evenness were observed across disease progression stages (S1-S4), neither in the haemolymph (Fig. 8B, C) nor the gut (Fig. 8E, F). Furthermore, no discernible clustering was observed in the PCoA based on the Bray–Curtis dissimilarity matrix in the haemolymph (Fig. 8D). However, a significant difference was detected in the gut microbiome data (PERMANOVA, *p* < 0.047; Fig. 8G). PERMDISP analysis confirmed no significant differences in multivariate dispersion across infection stages in the haemolymph (*p* = 0.7248) or the gut (*p* = 0.2323). Pairwise comparisons also showed no significant differences in dispersion among stages (all *p* > 0.05).

## Discussion

### Microbiome shifts associated with *Hematodinium* sp. infection

In the Clyde Sea Area, *Hematodinium* sp. prevalence is characterised by a seasonal patent infection peak that takes place in spring, although more sensitive diagnostic methods have shown that subpatent infection levels persist throughout the year [15]. Consistent with prior studies, our data indicate high infection levels in this area during this period. From a pathological perspective, early stages of infection are marked by limited changes in the number of haemocytes and a moderate presence of the parasite in tissues. However, advanced stages of infection exhibit significant parasite proliferation across all tissues, leading to metabolic exhaustion [21, 50]. Our data revealed that *Hematodinium* sp. infection has no discernible impact on bacterial diversity and evenness in either the gut or the haemolymph. However, a marked reduction in bacterial community richness was detected in the haemolymph. Although in general terms diversity was maintained, a distinct shift in the overall bacterial community composition was noted in both the gut and haemolymph of heavily infected individuals. Remarkably, this shift was already detected at very early stages of the subpatent infection (animals only positive by PCR). The occurrence of these changes at this very early stage and their continuance throughout more advanced stages suggest that the microbiome responds promptly to *Hematodinium* sp. infection, and these perturbations persist as the disease progresses.

Given that *Hematodinium* primarily inhabits and propagates in the haemolymph decreasing the haemocyte counts and turning it into a white milky fluid, alterations in the microbial community richness within the haemolymph were noticeable from the onset of the infection. A similar trend in richness reduction has been previously observed in shrimp with white faeces syndrome (WFS) [51, 52]. In addition, the overall bacterial community composition of haemolymph showed a marked dysbiosis, with differential genera analysis highlighting a significant reduction in the abundance of over 100 genera in the infected group. Of these, the genera *Blautia*, *Lachnospiraceae unclassified*, *Cellulosilyticum*, belong to the family *Lachnospiraceae*, were decreased in the infected group. *Lachnospiraceae* are essential producers of short-chain fatty acids, and they were described among the healthy microbiome of several species including the Chinese mitten crab (*Eriocheir sinensis*) [53, 54]. Likewise, *Peptostreptococcaceae* (*Acetoanaerobium*, *Intestinibacter*, *Paeniclostridium*, *Peptostreptococcaceae Unclassified*, *Romboutsia*) with their crucial metabolic capabilities were noticeably decreased [55]. On the contrary, the genera *Rhodovulum*, *Tessaracoccus*, *Chryseobacterium*, *Sphingobacterium*, *Wolinella*, *Rurimicrobium*, *Calditerrivibrio*, *Butyrivibrio*, *Crenothrix*, *Hydrogenophilus* and *Xanthomonas* were found to be abundant in *Hematodinium*-positive haemolymph. *Rhodovulum* has been associated with thermal stress response [56], while *Tessaracoccus* has been recently recognised as a potential human pathogen [57].

Despite the lack of significant changes in the alpha diversity indices of the gut microbiome, we noted a substantial shift in the overall gut community composition. This observation aligns with the findings of Wang and colleagues [58], who reported no significant differences in gut microbial richness and diversity during white spot syndrome virus (WSSV) infection in *Penaeus vannamei*. Nevertheless, they highlighted a marked difference in beta diversity, demonstrating a significant shift in the community composition. In contrast to haemolymph, over 80 genera showed a marked increase in abundance in the gut of infected *N. norvegicus*. Notably, in advanced stages of *Hematodinium* sp. infections, animals display lethargy and a state of decline, attributed to metabolic exhaustion and nutrient depletion induced by the parasite [23]. Despite their condition, heavily infected animals continue to eat, as evidenced by our observations of stomach contents being either full or partially filled. However, their diet may be more limited, with a decreased likelihood of behaving as predators and a greater reliance on plant-based and suspension feeding, potentially influenced by seasonal patterns and food availability. This shift in diet could explain the increased relative abundance of bacterial families capable of degrading complex polysaccharides (*Prevotellaceae*, *Bacteroidaceae, Muribaculaceae, Lachnospiraceae, Akkermansiaceae*) in infected individuals compared to non-infected ones. Interestingly, the continuous feeding behaviour of infected animals may also contribute to the lack of changes in microbial richness in the gut as the infection progresses. In addition, *Erysipelotrichaceae and Hydrogenophilaceae* were significantly higher in the gut of the infected group. The members of *Erysipelotrichaceae* (*Dubosiella*, *Faecalibaculum*, *Ileibacterium* and *Turicibacter*) exhibit high immunogenicity and are positively associated with host inflammation [59]. The higher abundance of *Hydrogenophilus* can be related to their capability to sustain growth under environmental stress and nutrient deprivation [60]. On the contrary, the abundance of the genera *Desulforhopalus, Lentisphaeria unclassified, Desulfovibrionaceae unclassified, Peptoclostridium,* and *P.palmC41 ge* showed a significant reduction in *Hematodinium* infected *N. norvegicus*. *Peptoclostridium* is involved in carbohydrate fermentation, whereas *Desulforhopalus* and *Desulfovibrionaceae are* involved in sulphur metabolism [61]. Changes in the abundance of these genera support the hypothesis that infected animals rely more on detritivores and suspensivores diet.

The crosstalk between the immune system and the microbiome is crucial for eliciting protective responses against pathogens including parasites. The host immune response to *Hematodinium* sp*.* infection is yet debatable. Immunomodulatory effects at both post-transcriptional and translational levels of haemocytes were detected following the challenge of the Chinese swimming crab (*Portunus trituberculatus*) with *Hematodinium perezi* [62]. In contrast, the *Hematodinium*-infected shore crabs (*Carcinus maenas*) disease progression did not lead to an increase in collateral infections and no evidence of haemocyte reactivity toward infection was detected, indicating the absence of a cellular-driven immune response at both early and advanced stages of infection [24]. Similarly, our histology data indicate the lack of host reactivity against *Hematodinium* sp. The onset and progression of parasitic infection are shaped by the dynamic interplay among the microbiota, parasite, and the host's immune system [63]. While deciphering this complex interaction during such infection can be challenging, it is possible that reported changes in the immune response in the haemolymph might be triggered by shifts in the microbiome. Besides supporting host immunity, the microbiome can directly influence the virulence of parasites [64]. Thus, microbial community imbalance (dysbiosis) could be a driver or predisposing factor to infection.

### Impact of size on haemolymph and gut microbiomes of *N. norvegicus*

Several studies have reported that small-sized *N. norvegicus* exhibit increased susceptibility to *Hematodinium* sp. infection [11, 13, 15]; a trend reported not only for this decapod species and parasite system as reviewed by [65]. This susceptibility is further supported by our recent observation demonstrating that smaller animals are more prone to developing advanced stages of infection [17]. The correlation between size and susceptibility raises intriguing questions about the underlying mechanisms. One significant finding of this study was the decreased richness of the haemolymph microbial community in smaller animals (25.2 ± 1.20 mm CL). This finding suggests a potential link between microbiome richness and susceptibility to infection. A less diverse microbial community may lack the protective factors, thereby predisposing smaller animals to the development of full infection. However, disease challenge experiments following disease progression in animals of different sizes would be required to further test this hypothesis. While our results offer valuable insights, they also raise additional questions. For instance, further studies are needed to identify specific microbial species and functional pathways associated with increased susceptibility of small animals to *Hematodinium* sp. infection.

### Haemolymph and gut microbiomes of *N. norvegicus*

This is the first study to provide an in-depth characterisation of the haemolymph and gut microbiomes of *N. norvegicus*. Employing high throughput 16S rRNA gene amplicon sequencing, we sought to reveal the taxonomic composition, core communities and shared taxa inhabiting the haemolymph and gut of apparent healthy *Hematodinium* sp. free *N. norvegicus*. Our data highlighted that the haemolymph harbours a highly rich, diverse, and distinct community compared to the gut. *N. norvegicus* possesses a semi-closed circulatory system, facilitating direct contact between haemolymph and internal organs. This direct contact could potentially serve as the source of the observed diversity and shared taxa with the gut. Microbiome organ specificity was also evident in marine invertebrates, such as the mud crab (*Scylla paramamosain*) [66], suggesting that the haemolymph plays a role in selecting its diverse community.

*Proteobacteria* were found to be the dominant phylum in both the haemolymph and gut, with an estimated abundance of 66.48% in the haemolymph and 46.11% in the gut. These findings are consistent with previous studies in other crustaceans and aquatic invertebrates [67–71]. In addition, the phyla *Actinobacteria, Bacteroidetes, Firmicutes, Fusobacteria*, and *Verrucomicrobia* were present in both the haemolymph and the gut. These phyla are also typically reported across other crustacean species’ [58, 72–74]. *Proteobacteria* are known to form symbiotic associations with a wide range of hosts, including marine organisms. In the case of *N. norvegicus*, the haemolymph and gut provide suitable niches for colonisation by *Proteobacteria*. The feeding behaviour of *N. norvegicus* and their continuous exposure to the marine environment likely introduce a diverse array of microorganisms, including *Proteobacteria*. The ability of *Proteobacteria* to establish and persist in these environments suggests a mutualistic or commensal relationship. Furthermore, given that *N. norvegicus* is a benthic and burrowing decapod crustacean commonly found in muddy substrata, it is not surprising to find phyla commonly dominant in sediment samples, such as *Actinobacteria*, *Acidobacteria*, *Chloroflexi,* and *Firmicutes* [75, 76].

The core microbiome analysis of the haemolymph revealed the presence of potentially beneficial genera such as *Bifidobacterium*, *Lactobacillus*, *Faecalibacterium* and *Subdoligranulum.* These genera have been reported in the healthy gut microbiomes of crustaceans [77], and they are capable of producing enzymes that facilitate the fermentation of indigestible carbohydrates into short-chain fatty acids (SCFAs). SCFAs have been recognised for their anti-inflammatory and immunomodulatory properties. It is plausible that these bacterial genera serve a similar function in the haemolymph, with their presence indicating the potential for taxa translocation between the gut and haemolymph [66]. In addition, *Pseudoalteromonas* was identified within the core community, and it has been reported in the haemolymph of invertebrates that produce antimicrobials against pathogenic bacteria, such as *Vibrio* [78, 79]. Opportunistic aquatic pathogens such as *Pseudomonas, Aeromonas*, and *Acinetobacter* genera were also identified among core haemolymph genera. On the other hand, the core microbiome of the gut encompasses a wide range of genera, including those associated with marine and anaerobic environments, as well as potential pathogens. Among these are sulfate-reducing bacteria such as the genera *Desulfatiferula*, *Desulfobulbaceae unclassified*, *Desulforhopalus*, *Desulfotalea*, and *Desulfovibrionaceae unclassified*. These bacteria have a potential role in the digestive process and overall health. The genus *Carboxylicivirga*, typically found in marine sediment [80], was identified as a part of the core gut community in this study. Notably, it was also reported within the core gut microbiomes of the mud crab (*Scylla paramamosain)* [81], and the European lobster (*Homarus gammarus)* [31]. *Photobacterium*, *Aliivibrio*, and *Shewanella*, belonging to the *Proteobacteria*, are commonly found among the core gut community of aquatic invertebrates, with some species known to be opportunistic pathogens. *Psychrilyobacter*, a genus of bacteria that utilises sugars, amino acids, and peptone as carbon sources, was identified within the core gut microbiome. Likewise, it was described as a dominant genus in the gut microbiome of deep-sea hydrothermal vent crab *Austinograe* sp. [82] and *Sycilla paramamosain* [81]. *Psychrilyobacter* produces H_2_ and acetate as primary fermentation by-products, suggesting its potential role in facilitating adaptation to hypoxic conditions [82]. In agreement with the meta-analysis of core gut bacteria of decapod crustaceans conducted by Foysal [83], *Candidatus Bacilloplasma* was among the core gut community. Duan and colleagues [84] reported the dominance of *Candidatus Bacilloplasma* in the healthy intestine of *Penaeus vannamei*, and their positive correlation with several pattern recognition receptors, highlighting the potential influence on the host immune responses.

In conclusion, the current study presents the first comprehensive high-throughput characterisation of the haemolymph and gut microbiomes of 'healthy' *N.  norvegicus*, elucidating the complex taxonomic composition and core communities. Moreover, it explores the dynamics of haemolymph and gut microbiomes during the course of *Hematodinium* infection. The evidence from this study emphasises the significant impact of infection on microbiomes, marked by shifts in the bacterial richness of the haemolymph and potential dysbiosis. These changes, observed at the early stages of infection, indicate a prompt and persistent microbiome response to the parasitic infection. The alterations in microbial community composition open up avenues to explore the potential role of the microbiome during host-parasite interaction. However, the underlying mechanisms of these observed changes remain to be explored, necessitating a more detailed investigation into the functional roles and interactions of the identified microbiota, as well as the host’s immune response, to fully understand the complex dynamics that characterise the interplay between host, microbiome, and *Hematodinium* sp.

## Supplementary Information

Additional file 1.

Additional file 2.

## Data Availability

Bacterial 16S rRNA Amplicon data: SRA submission is: SUB13729042 and BioProject ID is: PRJNA1001905.
