# Supplementary material for: Microbiome dynamics associated with Hematodinium sp. infection in Norway lobster (Nephrops norvegicus)
Source: Anim Microbiome. 2025 Jun 13;7:62. doi: 10.1186/s42523-025-00416-w (PMC12164141; doi:10.1186/s42523-025-00416-w)
Supplement: Supplementary file 1 — Additional file 1. [file 42523_2025_416_MOESM1_ESM.docx]

**Supplementary Table S1.** List of the shared taxa between the haemolymph and gut of apparent healthy *Nephrops norvegicus* (tested negative for *Hematodinium* sp. by BCM, PM, PCR, histology, n=45).

| OTU | Taxonomy |
| --- | --- |
| OTU 29 | *Proteobacteria*; *Gammaproteobacteria*; *Enterobacteriales*; *Enterobacteriaceae*; *Enterobacteriaceae unclassified* |
| OTU 40 | *Proteobacteria*; *Gammaproteobacteria*; *Enterobacteriales*; *Enterobacteriaceae*; *Enterobacteriaceae unclassified* |
| OTU 56 | *Proteobacteria*; *Gammaproteobacteria*; *Enterobacteriales*; *Enterobacteriaceae*; *Enterobacteriaceae unclassified* |
| OTU 158 | *Proteobacteria*; *Gammaproteobacteria*; *Enterobacteriales*; *Enterobacteriaceae*; *Enterobacteriaceae unclassified* |
| OTU 192 | *Proteobacteria*; *Gammaproteobacteria*; *Enterobacteriales*; *Enterobacteriaceae*; *Enterobacteriaceae unclassified* |
| OTU 314 | *Proteobacteria*; *Gammaproteobacteria*; *Enterobacteriales*; *Enterobacteriaceae*; *Enterobacteriaceae unclassified* |
| OTU 395 | *Proteobacteria*; *Gammaproteobacteria*; *Enterobacteriales*; *Enterobacteriaceae*; *Enterobacteriaceae unclassified* |
| OTU 407 | *Proteobacteria*; *Gammaproteobacteria*; *Enterobacteriales*; *Enterobacteriaceae*; *Enterobacteriaceae unclassified* |
| OTU 425 | *Proteobacteria*; *Gammaproteobacteria*; *Pseudomonadales*; *Pseudomonadaceae*; *Pseudomonas* |
| OTU 479 | *Proteobacteria*; *Gammaproteobacteria*; *Enterobacteriales*; *Enterobacteriaceae*; *Enterobacteriaceae unclassified* |
| OTU 585 | *Bacteroidetes*; *Bacteroidia*; *Bacteroidales*; *Muribaculaceae*; *Muribaculaceae ge* |
| OTU 731 | *Proteobacteria*; *Gammaproteobacteria*; *Enterobacteriales*; *Enterobacteriaceae*; *Enterobacteriaceae unclassified* |
| OTU 762 | *Bacteroidetes*; *Bacteroidia*; *Bacteroidales*; *Muribaculaceae*; *Muribaculaceae ge* |
| OTU 766 | *Proteobacteria*; *Gammaproteobacteria*; *Enterobacteriales*; *Enterobacteriaceae*; *Enterobacteriaceae unclassified* |
| OTU 790 | *Bacteroidetes*; *Bacteroidia*; *Bacteroidales*; *Muribaculaceae*; *Muribaculaceae ge* |
| OTU 1172 | *Proteobacteria*; *Gammaproteobacteria*; *Enterobacteriales*; *Enterobacteriaceae*; *Enterobacteriaceae unclassified* |
| OTU 1342 | *Firmicutes*; *Bacilli*; *Lactobacillales*; *Streptococcaceae*; *Streptococcus* |
| OTU 1366 | *Bacteroidetes*; *Bacteroidia*; *Bacteroidales*; *Muribaculaceae*; *Muribaculaceae ge* |
| OTU 1531 | *Proteobacteria*; *Gammaproteobacteria*; *Betaproteobacteriales*; *Burkholderiaceae*; *Ralstonia* |
| OTU 1642 | *Bacteroidetes*; *Bacteroidia*; *Bacteroidales*; *Muribaculaceae*; *Muribaculaceae ge* |
| OTU 1731 | *Bacteroidetes*; *Bacteroidia*; *Bacteroidales*; *Muribaculaceae*; *Muribaculaceae unclassified* |
| OTU 2437 | *Bacteroidetes*; *Bacteroidia*; *Bacteroidales*; *Muribaculaceae*; *Muribaculaceae ge* |
| OTU 2654 | *Firmicutes*; *Bacilli*; *Bacillales*; *Bacillaceae*; *Bacillaceae unclassified* |
| OTU 2790 | *Firmicutes*; *Clostridia*; *Clostridiales*; *Ruminococcaceae*; *Subdoligranulum* |
| OTU 2877 | *Firmicutes*; *Bacilli*; *Lactobacillales*; *Enterococcaceae*; *Enterococcaceae unclassified* |
| OTU 2923 | *Proteobacteria*; *Gammaproteobacteria*; *Pseudomonadales*; *Pseudomonadaceae*; *Pseudomonas* |
| OTU 2949 | *Proteobacteria*; *Gammaproteobacteria*; *Enterobacteriales*; *Enterobacteriaceae*; *Enterobacteriaceae unclassified* |
| OTU 2969 | *Bacteroidetes*; *Bacteroidia*; *Bacteroidales*; *Muribaculaceae*; *Muribaculaceae ge* |
| OTU 3293 | *Epsilonbacteraeota*; *Campylobacteria*; *Campylobacterales*; *Helicobacteraceae*; *Helicobacter* |
| OTU 3369 | *Firmicutes*; *Erysipelotrichia*; *Erysipelotrichales*; *Erysipelotrichaceae*; *Erysipelatoclostridium* |
| OTU 4034 | *Firmicutes*; *Clostridia*; *Clostridiales*; *Ruminococcaceae*; *Subdoligranulum* |

**Supplementary Table S2.** The core microbiome of haemolymph of apparent healthy *Nephrops norvegicus* (tested negative for *Hematodinium* sp. by BCM, PM, PCR, histology, n=45).

| Phylum | Class | Order | Family | Genus |
| --- | --- | --- | --- | --- |
| *Proteobacteria* | *Gammaproteobacteria* | *Gammaproteobacteria Incertae Sedis* | *Unknown Family* | *Acidibacter* |
| *Proteobacteria* | *Gammaproteobacteria* | *Pseudomonadales* | *Moraxellaceae* | *Acinetobacter* |
| *Bacteroidetes* | *Bacteroidia* | *Cytophagales* | *Hymenobacteraceae* | *Adhaeribacter* |
| *Proteobacteria* | *Gammaproteobacteria* | *Aeromonadales* | *Aeromonadaceae* | *Aeromonas* |
| *Firmicutes* | *Clostridia* | *Clostridiales* | *Lachnospiraceae* | *Agathobacter* |
| *Verrucomicrobia* | *Verrucomicrobiae* | *Verrucomicrobiales* | *Akkermansiaceae* | *Akkermansia* |
| *Proteobacteria* | *Gammaproteobacteria* | *Betaproteobacteriales* | *Burkholderiaceae* | *Alcaligenes* |
| *Proteobacteria* | *Gammaproteobacteria* | *Alteromonadales* | *Alteromonadaceae* | *Alishewanella* |
| *Bacteroidetes* | *Bacteroidia* | *Bacteroidales* | *Rikenellaceae* | *Alistipes* |
| *Bacteroidetes* | *Bacteroidia* | *Bacteroidales* | *Prevotellaceae* | *Alloprevotella* |
| *Proteobacteria* | *Gammaproteobacteria* | *Alteromonadales* | *Alteromonadaceae* | *Alteromonadaceae unclassified* |
| *Proteobacteria* | *Alphaproteobacteria* | *Rhodobacterales* | *Rhodobacteraceae* | *Amylibacter* |
| *Bacteroidetes* | *Bacteroidia* | *Flavobacteriales* | *Flavobacteriaceae* | *Aurantivirga* |
| *Firmicutes* | *Bacilli* | *Bacillales* | *Bacillaceae* | *Bacillaceae unclassified* |
| *Bacteroidetes* | *Bacteroidia* | *Bacteroidales* | *Bacteroidaceae* | *Bacteroides* |
| *Actinobacteria* | *Actinobacteria* | *Bifidobacteriales* | *Bifidobacteriaceae* | *Bifidobacterium* |
| *Firmicutes* | *Clostridia* | *Clostridiales* | *Lachnospiraceae* | *Blautia* |
| *Proteobacteria* | *Alphaproteobacteria* | *Caulobacterales* | *Caulobacteraceae* | *Brevundimonas* |
| *Proteobacteria* | *Gammaproteobacteria* | *Betaproteobacteriales* | *Burkholderiaceae* | *Burkholderiaceae unclassified* |
| *Proteobacteria* | *Alphaproteobacteria* | *Puniceispirillales* | *SAR116 clade* | *Candidatus Puniceispirillum* |
| *Proteobacteria* | *Gammaproteobacteria* | *Thiomicrospirales* | *Thioglobaceae* | *Candidatus Thioglobus* |
| *Firmicutes* | *Clostridia* | *Clostridiales* | *Lachnospiraceae* | *Catonella* |
| *Proteobacteria* | *Alphaproteobacteria* | *Caulobacterales* | *Caulobacteraceae* | *Caulobacter* |
| *Proteobacteria* | *Alphaproteobacteria* | *Caulobacterales* | *Caulobacteraceae* | *Caulobacteraceae unclassified* |
| *Bacteroidetes* | *Bacteroidia* | *Flavobacteriales* | *Weeksellaceae* | *Chryseobacterium* |
| *Proteobacteria* | *Alphaproteobacteria* | *SAR11 clade* | *Clade I* | *Clade Ia* |
| *Actinobacteria* | *Actinobacteria* | *Corynebacteriales* | *Corynebacteriaceae* | *Corynebacteriaceae unclassified* |
| *Actinobacteria* | *Actinobacteria* | *Propionibacteriales* | *Propionibacteriaceae* | *Cutibacterium* |
| *Proteobacteria* | *Deltaproteobacteria* | *Desulfovibrionales* | *Desulfovibrionaceae* | *Desulfovibrio* |
| *Bacteroidetes* | *Bacteroidia* | *Bacteroidales* | *Dysgonomonadaceae* | *Dysgonomonas* |
| *Bacteroidetes* | *Bacteroidia* | *Flavobacteriales* | *Weeksellaceae* | *Elizabethkingia* |
| *Proteobacteria* | *Gammaproteobacteria* | *Pseudomonadales* | *Moraxellaceae* | *Enhydrobacter* |
| *Proteobacteria* | *Gammaproteobacteria* | *Enterobacteriales* | *Enterobacteriaceae* | *Enterobacteriaceae unclassified* |
| *Firmicutes* | *Bacilli* | *Lactobacillales* | *Enterococcaceae* | *Enterococcaceae unclassified* |
| *Firmicutes* | *Erysipelotrichia* | *Erysipelotrichales* | *Erysipelotrichaceae* | *Erysipelatoclostridium* |
| *Firmicutes* | *Erysipelotrichia* | *Erysipelotrichales* | *Erysipelotrichaceae* | *Erysipelotrichaceae ge* |
| *Firmicutes* | *Clostridia* | *Clostridiales* | *Ruminococcaceae* | *Faecalibacterium* |
| *Firmicutes* | *Clostridia* | *Clostridiales* | *Family XIII* | *Family XIII ge* |
| *Firmicutes* | *Clostridia* | *Clostridiales* | *Peptostreptococcaceae* | *Filifactor* |
| *Bacteroidetes* | *Bacteroidia* | *Flavobacteriales* | *Flavobacteriaceae* | *Flavobacteriaceae unclassified* |
| *Bacteroidetes* | *Bacteroidia* | *Flavobacteriales* | *Crocinitomicaceae* | *Fluviicola* |
| *Proteobacteria* | *Gammaproteobacteria* | *Gammaproteobacteria unclassified* | *Gammaproteobacteria unclassified* | *Gammaproteobacteria unclassified* |
| *Firmicutes* | *Bacilli* | *Bacillales* | *Family XI* | *Gemella* |
| *Proteobacteria* | *Gammaproteobacteria* | *Alteromonadales* | *Alteromonadaceae* | *Glaciecola* |
| *Firmicutes* | *Bacilli* | *Lactobacillales* | *Carnobacteriaceae* | *Granulicatella* |
| *Proteobacteria* | *Gammaproteobacteria* | *Cellvibrionales* | *Halieaceae* | *Halieaceae unclassified* |
| *Proteobacteria* | *Gammaproteobacteria* | *Oceanospirillales* | *Halomonadaceae* | *Halomonadaceae unclassified* |
| *Epsilonbacteraeota* | *Campylobacteria* | *Campylobacterales* | *Helicobacteraceae* | *Helicobacter* |
| *Firmicutes* | *Clostridia* | *Clostridiales* | *Peptostreptococcaceae* | *Intestinibacter* |
| *Actinobacteria* | *Actinobacteria* | *Micrococcales* | *Intrasporangiaceae* | *Intrasporangiaceae unclassified* |
| *Firmicutes* | *Clostridia* | *Clostridiales* | *Lachnospiraceae* | *Lachnospiraceae ge* |
| *Firmicutes* | *Clostridia* | *Clostridiales* | *Lachnospiraceae* | *Lachnospiraceae unclassified* |
| *Firmicutes* | *Bacilli* | *Lactobacillales* | *Lactobacillales unclassified* | *Lactobacillales unclassified* |
| *Firmicutes* | *Bacilli* | *Lactobacillales* | *Lactobacillaceae* | *Lactobacillus* |
| *Firmicutes* | *Bacilli* | *Lactobacillales* | *Leuconostocaceae* | *Leuconostoc* |
| *Proteobacteria* | *Alphaproteobacteria* | *Rhodobacterales* | *Rhodobacteraceae* | *Litoreibacter* |
| *Proteobacteria* | *Alphaproteobacteria* | *Rhodobacterales* | *Rhodobacteraceae* | *Marivivens* |
| *Firmicutes* | *Negativicutes* | *Selenomonadales* | *Veillonellaceae* | *Megasphaera* |
| *Proteobacteria* | *Gammaproteobacteria* | *Nitrosococcales* | *Methylophagaceae* | *Methylophagaceae unclassified* |
| *Proteobacteria* | *Gammaproteobacteria* | *Betaproteobacteriales* | *Methylophilaceae* | *Methylophilaceae unclassified* |
| *Proteobacteria* | *Gammaproteobacteria* | *Betaproteobacteriales* | *Methylophilaceae* | *Methylophilaceae unclassified* |
| *Proteobacteria* | *Alphaproteobacteria* | *Micropepsales* | *Micropepsaceae* | *Micropepsaceae unclassified* |
| *Bacteroidetes* | *Bacteroidia* | *Bacteroidales* | *Muribaculaceae* | *Muribaculaceae ge* |
| *Bacteroidetes* | *Bacteroidia* | *Bacteroidales* | *Muribaculaceae* | *Muribaculaceae unclassified* |
| *Proteobacteria* | *Gammaproteobacteria* | *Betaproteobacteriales* | *Neisseriaceae* | *Neisseria* |
| *Proteobacteria* | *Gammaproteobacteria* | *Betaproteobacteriales* | *Neisseriaceae* | *Neisseriaceae unclassified* |
| *Proteobacteria* | *Gammaproteobacteria* | *Salinisphaerales* | *Solimonadaceae* | *Nevskia* |
| *Bacteroidetes* | *Bacteroidia* | *Sphingobacteriales* | *NS11-12 marine group* | *NS11-12 marine group ge* |
| *Bacteroidetes* | *Bacteroidia* | *Flavobacteriales* | *Flavobacteriaceae* | *NS4 marine group* |
| *Bacteroidetes* | *Bacteroidia* | *Flavobacteriales* | *Flavobacteriaceae* | *NS5 marine group* |
| *Proteobacteria* | *Gammaproteobacteria* | *Betaproteobacteriales* | *Methylophilaceae* | *OM43 clade* |
| *Proteobacteria* | *Gammaproteobacteria* | *Cellvibrionales* | *Halieaceae* | *OM60(NOR5) clade* |
| *Firmicutes* | *Clostridia* | *Clostridiales* | *Lachnospiraceae* | *Oribacterium* |
| *Firmicutes* | *Clostridia* | *Clostridiales* | *Peptostreptococcaceae* | *Paeniclostridium* |
| *Bacteroidetes* | *Bacteroidia* | *Bacteroidales* | *Tannerellaceae* | *Parabacteroides* |
| *Proteobacteria* | *Gammaproteobacteria* | *Pasteurellales* | *Pasteurellaceae* | *Pasteurellaceae unclassified* |
| *Bacteroidetes* | *Bacteroidia* | *Flavobacteriales* | *Flavobacteriaceae* | *Polaribacter 3* |
| *Proteobacteria* | *Gammaproteobacteria* | *Cellvibrionales* | *Porticoccaceae* | *Porticoccaceae unclassified* |
| *Bacteroidetes* | *Bacteroidia* | *Bacteroidales* | *Prevotellaceae* | *Prevotella 7* |
| *Bacteroidetes* | *Bacteroidia* | *Bacteroidales* | *Prevotellaceae* | *Prevotella 9* |
| *Proteobacteria* | *Gammaproteobacteria* | *Alteromonadales* | *Pseudoalteromonadaceae* | *Pseudoalteromonas* |
| *Proteobacteria* | *Gammaproteobacteria* | *Oceanospirillales* | *Pseudohongiellaceae* | *Pseudohongiella* |
| *Proteobacteria* | *Gammaproteobacteria* | *Pseudomonadales* | *Pseudomonadaceae* | *Pseudomonadaceae unclassified* |
| *Proteobacteria* | *Gammaproteobacteria* | *Pseudomonadales* | *Pseudomonadaceae* | *Pseudomonas* |
| *Proteobacteria* | *Gammaproteobacteria* | *Betaproteobacteriales* | *Burkholderiaceae* | *Ralstonia* |
| *Proteobacteria* | *Alphaproteobacteria* | *Rhizobiales* | *Rhizobiaceae* | *Rhizobiaceae unclassified* |
| *Proteobacteria* | *Alphaproteobacteria* | *Rhodobacterales* | *Rhodobacteraceae* | *Rhodobacteraceae unclassified* |
| *Firmicutes* | *Clostridia* | *Clostridiales* | *Peptostreptococcaceae* | *Romboutsia* |
| *Firmicutes* | *Clostridia* | *Clostridiales* | *Lachnospiraceae* | *Roseburia* |
| *Firmicutes* | *Clostridia* | *Clostridiales* | *Ruminococcaceae* | *Ruminococcaceae UCG-014* |
| *Proteobacteria* | *Alphaproteobacteria* | *Puniceispirillales* | *SAR116 clade* | *SAR116 clade ge* |
| *Proteobacteria* | *Gammaproteobacteria* | *Cellvibrionales* | *Porticoccaceae* | *SAR92 clade* |
| *Firmicutes* | *Erysipelotrichia* | *Erysipelotrichales* | *Erysipelotrichaceae* | *Solobacterium* |
| *Firmicutes* | *Bacilli* | *Bacillales* | *Staphylococcaceae* | *Staphylococcus* |
| *Proteobacteria* | *Gammaproteobacteria* | *Xanthomonadales* | *Xanthomonadaceae* | *Stenotrophomonas* |
| *Firmicutes* | *Clostridia* | *Clostridiales* | *Lachnospiraceae* | *Stomatobaculum* |
| *Firmicutes* | *Bacilli* | *Lactobacillales* | *Streptococcaceae* | *Streptococcus* |
| *Firmicutes* | *Clostridia* | *Clostridiales* | *Ruminococcaceae* | *Subdoligranulum* |
| *Acidobacteria* | *Acidobacteriia* | *Subgroup 2* | *Subgroup 2 fa* | *Subgroup 2 ge* |
| *Proteobacteria* | *Gammaproteobacteria* | *Aeromonadales* | *Succinivibrionaceae* | *Succinivibrionaceae unclassified* |
| *Proteobacteria* | *Gammaproteobacteria* | *Thiomicrospirales* | *Thioglobaceae* | *SUP05 cluster* |
| *Proteobacteria* | *Gammaproteobacteria* | *Thiomicrospirales* | *Thioglobaceae* | *Thioglobaceae unclassified* |
| *Proteobacteria* | *Alphaproteobacteria* | *Caulobacterales* | *Caulobacteraceae* | *uncultured* |
| *Bacteroidetes* | *Bacteroidia* | *Flavobacteriales* | *Cryomorphaceae* | *uncultured* |
| *Proteobacteria* | *Deltaproteobacteria* | *Desulfovibrionales* | *Desulfovibrionaceae* | *uncultured* |
| *Proteobacteria* | *Gammaproteobacteria* | *Ectothiorhodospirales* | *Ectothiorhodospiraceae* | *uncultured* |
| *Proteobacteria* | *Gammaproteobacteria* | *Nitrosococcales* | *Methylophagaceae* | *uncultured* |
| *Acidobacteria* | *Acidobacteriia* | *Acidobacteriales* | *uncultured* | *uncultured ge* |
| *Firmicutes* | *Negativicutes* | *Selenomonadales* | *Veillonellaceae* | *Veillonella* |
| *Proteobacteria* | *Gammaproteobacteria* | *Vibrionales* | *Vibrionaceae* | *Vibrionaceae unclassified* |
| *Bacteroidetes* | *Bacteroidia* | *Chitinophagales* | *Chitinophagaceae* | *Vibrionimonas* |
| *Proteobacteria* | *Gammaproteobacteria* | *WD260* | *WD260 fa* | *WD260 ge* |
| *Bacteroidetes* | *Bacteroidia* | *Flavobacteriales* | *Weeksellaceae* | *Weeksellaceae unclassified* |
| *Proteobacteria* | *Alphaproteobacteria* | *Rhizobiales* | *Xanthobacteraceae* | *Xanthobacteraceae unclassified* |
| *Proteobacteria* | *Gammaproteobacteria* | *Xanthomonadales* | *Xanthomonadaceae* | *Xanthomonas* |

**Supplementary Table S3.** The core microbiome of the gut of apparent healthy *Nephrops norvegicus* (tested negative for *Hematodinium* sp. by BCM, PM, PCR, histology, n=45).

| Phylum | Class | Order | Family | Genus |
| --- | --- | --- | --- | --- |
| *Proteobacteria* | *Gammaproteobacteria* | *Vibrionales* | *Vibrionaceae* | *Aliivibrio* |
| *Proteobacteria* | *Alphaproteobacteria* | *Alphaproteobacteria unclassified* | *Alphaproteobacteria unclassified* | *Alphaproteobacteria unclassified* |
| *Epsilonbacteraeota* | *Campylobacteria* | *Campylobacterales* | *Arcobacteraceae* | *Arcobacter* |
| *Bacteria unclassified* | *Bacteria unclassified* | *Bacteria unclassified* | *Bacteria unclassified* | *Bacteria unclassified* |
| *Bacteroidetes* | *Bacteroidia* | *Bacteroidia unclassified* | *Bacteroidia unclassified* | *Bacteroidia unclassified* |
| *Proteobacteria* | *Gammaproteobacteria* | *Betaproteobacteriales* | *Burkholderiaceae* | *Burkholderiaceae unclassified* |
| *Tenericutes* | *Mollicutes* | *Mycoplasmatales* | *Mycoplasmataceae* | *Candidatus Bacilloplasma* |
| *Tenericutes* | *Mollicutes* | *Entomoplasmatales* | *Entomoplasmatales Incertae Sedis* | *Candidatus Hepatoplasma* |
| *Bacteroidetes* | *Bacteroidia* | *Bacteroidales* | *Marinilabiliaceae* | *Carboxylicivirga* |
| *Firmicutes* | *Clostridia* | *Clostridiales* | *Clostridiaceae 1* | *Clostridiaceae 1 unclassified* |
| *Proteobacteria* | *Deltaproteobacteria* | *Desulfobacterales* | *Desulfobacteraceae* | *Desulfatiferula* |
| *Proteobacteria* | *Deltaproteobacteria* | *Desulfobacterales* | *Desulfobulbaceae* | *Desulfobulbaceae unclassified* |
| *Proteobacteria* | *Deltaproteobacteria* | *Desulfobacterales* | *Desulfobulbaceae* | *Desulforhopalus* |
| *Proteobacteria* | *Deltaproteobacteria* | *Desulfobacterales* | *Desulfobulbaceae* | *Desulfotalea* |
| *Proteobacteria* | *Deltaproteobacteria* | *Desulfovibrionales* | *Desulfovibrionaceae* | *Desulfovibrionaceae unclassified* |
| *Proteobacteria* | *Gammaproteobacteria* | *Enterobacteriales* | *Enterobacteriaceae* | *Enterobacteriaceae unclassified* |
| *Bacteroidetes* | *Bacteroidia* | *Flavobacteriales* | *Flavobacteriaceae* | *Flavobacteriaceae unclassified* |
| *Proteobacteria* | *Gammaproteobacteria* | *Gammaproteobacteria unclassified* | *Gammaproteobacteria unclassified* | *Gammaproteobacteria unclassified* |
| *Bacteroidetes* | *Bacteroidia* | *Bacteroidales* | *Marinifilaceae* | *Marinifilum* |
| *Lentisphaerae* | *Oligosphaeria* | *P.palmC41* | *P.palmC41 fa* | *P.palmC41 ge* |
| *Firmicutes* | *Clostridia* | *Clostridiales* | *Peptostreptococcaceae* | *Peptoclostridium* |
| *Proteobacteria* | *Gammaproteobacteria* | *Vibrionales* | *Vibrionaceae* | *Photobacterium* |
| *Fusobacteria* | *Fusobacteriia* | *Fusobacteriales* | *Fusobacteriaceae* | *Psychrilyobacter* |
| *Proteobacteria* | *Gammaproteobacteria* | *Alteromonadales* | *Psychromonadaceae* | *Psychromonas* |
| *Proteobacteria* | *Gammaproteobacteria* | *Alteromonadales* | *Shewanellaceae* | *Shewanella* |
| *Spirochaetes* | *Spirochaetia* | *Spirochaetales* | *Spirochaetaceae* | *Spirochaeta 2* |
| *Spirochaetes* | *Spirochaetia* | *Spirochaetales* | *Spirochaetaceae* | *Spirochaetaceae unclassified* |
| *Bacteroidetes* | *Bacteroidia* | *Flavobacteriales* | *Flavobacteriaceae* | *Spongiimonas* |
| *Proteobacteria* | *Gammaproteobacteria* | *Xanthomonadales* | *Xanthomonadaceae* | *Stenotrophomonas* |
| *Firmicutes* | *Bacilli* | *Lactobacillales* | *Streptococcaceae* | *Streptococcus* |
| *Epsilonbacteraeota* | *Campylobacteria* | *Campylobacterales* | *Sulfurospirillaceae* | *Sulfurospirillum* |
| *Proteobacteria* | *Gammaproteobacteria* | *Vibrionales* | *Vibrionaceae* | *Vibrionaceae unclassified* |
| *Firmicutes* | *Erysipelotrichia* | *Erysipelotrichales* | *Erysipelotrichaceae* | *ZOR0006* |

**Supplementary Table S4**. List of significantly different genera in the haemolymph microbiome of *Nephrops norvegicus* between *'Hematodinium*-free' (Negative by BCM, PM, PCR, and Histology; n=22) and *Hematodinium* positive (Positive by BCM, Pleopod Stages 3 and 4; n=22) (Wilcoxon rank-sum, *p* < 0.05).

| Taxonomy | | | Log 10 mean relative abundance | | Adjusted p-value |
| --- | --- | --- | --- | --- | --- |
| Phylum | **Family** | **Genus** | **Negative** | **Positive** |  |
| *Acidobacteriota* | *Acidobacteriaceae*  *(Subgroup 1)* | *Acidipila* | 3.20E-04 | 4.89E-05 | 2.37E-02 |
| *Acidobacteriota* | *Acidobacteriae*  *unclassified* | *Acidobacteriae*  *unclassified* | 2.01E-04 | 7.11E-05 | 6.96E-03 |
| *Acidobacteriota* | *Bryobacteraceae* | *Bryobacter* | 6.92E-04 | 2.66E-04 | 1.07E-02 |
| *Acidobacteriota* | *Solibacteraceae* | *Candidatus Solibacter* | 6.47E-04 | 1.69E-04 | 1.26E-02 |
| *Acidobacteriota* | *Acidobacteriaceae (Subgroup 1)* | *Granulicella* | 4.37E-04 | 1.25E-04 | 6.65E-03 |
| *Acidobacteriota* | *Acidobacteriaceae (Subgroup 1)* | *Occallatibacter* | 9.70E-05 | 3.38E-05 | 1.37E-02 |
| *Acidobacteriota* | *Blastocatellaceae* | *Stenotrophobacter* | 3.21E-05 | 3.44E-06 | 6.65E-03 |
| *Acidobacteriota* | *Subgroup 12 fa* | *Subgroup 12 ge* | 1.82E-05 | 9.78E-06 | 3.32E-02 |
| *Acidobacteriota* | *Subgroup 13 fa* | *Subgroup 13 ge* | 1.13E-04 | 9.81E-06 | 6.65E-03 |
| *Acidobacteriota* | *Subgroup 15 fa* | *Subgroup 15 ge* | 7.63E-05 | 1.53E-05 | 8.92E-03 |
| *Acidobacteriota* | *Subgroup 18 fa* | *Subgroup 18 ge* | 5.94E-05 | 1.84E-05 | 4.08E-02 |
| *Acidobacteriota* | *Subgroup 2 fa* | *Subgroup 2 ge* | 2.43E-03 | 3.62E-04 | 1.64E-02 |
| *Acidobacteriota* | *Subgroup 21 fa* | *Subgroup 21 ge* | 7.48E-05 | 2.13E-05 | 3.08E-02 |
| *Acidobacteriota* | *Subgroup 25 fa* | *Subgroup 25 ge* | 3.89E-05 | 1.45E-05 | 4.51E-02 |
| *Acidobacteriota* | *Subgroup 5 fa* | *Subgroup 5 ge* | 1.80E-04 | 3.85E-05 | 6.65E-03 |
| *Acidobacteriota* | *Subgroup 7 fa* | *Subgroup 7 ge* | 5.60E-04 | 2.41E-04 | 4.28E-02 |
| *Acidobacteriota* | *Subgroup 9 fa* | *Subgroup 9 ge* | 3.21E-05 | 5.62E-06 | 3.72E-02 |
| *Acidobacteriota* | *Vicinamibacteraceae* | *Vicinamibacter* | 4.58E-05 | 2.56E-06 | 1.81E-02 |
| *Acidobacteriota* | *Vicinamibacteraceae* | *Vicinamibacteraceae ge* | 7.94E-04 | 3.44E-04 | 3.47E-02 |
| *Actinobacteriota* | *Acidothermaceae* | *Acidothermus* | 6.91E-04 | 1.27E-04 | 4.08E-02 |
| *Actinobacteriota* | *Micromonosporaceae* | *Actinoplanes* | 6.68E-05 | 1.30E-05 | 1.90E-02 |
| *Actinobacteriota* | *Micromonosporaceae* | *Dactylosporangium* | 4.29E-05 | 1.18E-06 | 1.07E-02 |
| *Actinobacteriota* | *Demequinaceae* | *Demequina* | 1.05E-05 | 6.22E-07 | 4.69E-02 |
| *Actinobacteriota* | *Demequinaceae* | *Demequinaceae*  *unclassified* | 6.28E-05 | 5.43E-06 | 3.59E-02 |
| *Actinobacteriota* | *Eggerthellaceae* | *Eggerthella* | 1.81E-04 | 8.70E-05 | 4.37E-02 |
| *Actinobacteriota* | *Gaiellales unclassified* | *Gaiellales unclassified* | 1.98E-04 | 8.48E-05 | 3.72E-02 |
| *Actinobacteriota* | *IMCC26256 fa* | *IMCC26256 ge* | 3.62E-04 | 1.38E-04 | 3.47E-02 |
| *Actinobacteriota* | *Frankiaceae* | *Jatrophihabitans* | 1.22E-04 | 5.61E-05 | 4.33E-02 |
| *Actinobacteriota* | *Microbacteriaceae* | *Leucobacter* | 4.28E-05 | 3.07E-05 | 4.99E-02 |
| *Actinobacteriota* | *Intrasporangiaceae* | *Oryzihumus* | 4.52E-05 | 0.00E+00 | 3.08E-02 |
| *Actinobacteriota* | *Solirubrobacteraceae* | *Patulibacter* | 1.11E-05 | 1.65E-06 | 4.37E-02 |
| *Actinobacteriota* | *Eggerthellaceae* | *Slackia* | 2.11E-05 | 1.03E-05 | 4.33E-02 |
| *Actinobacteriota* | *Propionibacteriaceae* | *Tessaracoccus* | 0.00E+00 | 3.59E-05 | 4.33E-02 |
| *Bacteroidota* | *Muribaculaceae* | *CAG-873* | 2.31E-05 | 8.71E-05 | 3.72E-02 |
| *Bacteroidota* | *Weeksellaceae* | *Chryseobacterium* | 1.10E-03 | 4.06E-03 | 3.08E-02 |
| *Bacteroidota* | *Melioribacteraceae* | *IheB3-7* | 4.64E-05 | 9.22E-06 | 4.33E-02 |
| *Bacteroidota* | *Microscillaceae* | *Microscillaceae unclassified* | 1.08E-04 | 3.48E-05 | 4.46E-02 |
| *Bacteroidota* | *Chitinophagaceae* | *Niastella* | 3.32E-04 | 2.29E-05 | 6.65E-03 |
| *Bacteroidota* | *Saprospiraceae* | *Phaeodactylibacter* | 8.04E-05 | 2.70E-05 | 2.27E-02 |
| *Bacteroidota* | *Chitinophagaceae* | *Rurimicrobium* | 0.00E+00 | 1.25E-05 | 2.03E-02 |
| *Bacteroidota* | *Rikenellaceae* | *S50 wastewater-sludge group* | 3.22E-05 | 9.25E-06 | 4.83E-02 |
| *Bacteroidota* | *Rhodothermaceae* | *Salinibacter* | 4.16E-05 | 0.00E+00 | 4.33E-02 |
| *Bacteroidota* | *Sphingobacteriaceae* | *Sphingobacterium* | 2.57E-04 | 7.20E-04 | 2.86E-02 |
| *Calditrichota* | *Calditrichaceae* | *Calditrichaceae unclassified* | 4.19E-05 | 2.65E-06 | 6.65E-03 |
| *Campilobacterota* | *Helicobacteraceae* | *Wolinella* | 3.13E-07 | 2.86E-05 | 2.50E-02 |
| *Chloroflexi* | *Ktedonobacteraceae* | *1959-1* | 1.23E-05 | 0.00E+00 | 4.33E-02 |
| *Chloroflexi* | *Herpetosiphonaceae* | *Herpetosiphon* | 1.61E-05 | 0.00E+00 | 2.03E-02 |
| *Chloroflexi* | *JG30-KF-AS9* | *JG30-KF-AS9 ge* | 7.39E-05 | 1.47E-05 | 6.65E-03 |
| *Chloroflexi* | *KD4-96 fa* | *KD4-96 ge* | 4.58E-04 | 2.22E-04 | 2.53E-02 |
| *Cyanobacteria* | *Unknown Family* | *Leptolyngbya ANT.L52.2* | 2.07E-05 | 3.69E-07 | 1.62E-02 |
| *Cyanobacteria* | *Coleofasciculaceae* | *Microcoleus SAG 1449-1a* | 3.25E-05 | 0.00E+00 | 2.03E-02 |
| *Cyanobacteria* | *Sericytochromatia fa* | *Sericytochromatia*  *ge* | 2.09E-05 | 1.42E-06 | 2.10E-02 |
| *Deferribacterota* | *Deferribacteraceae* | *Calditerrivibrio* | 0.00E+00 | 7.95E-06 | 3.08E-02 |
| *Deinococcota* | *Thermaceae* | *Meiothermus* | 2.07E-05 | 1.65E-06 | 1.62E-02 |
| *Desulfobacterota* | *Desulfuromonadaceae* | *Desulfuromonadaceae*  *unclassified* | 1.82E-05 | 3.59E-06 | 2.03E-02 |
| *Entotheonellaeota* | *Entotheonellaceae* | *Entotheonellaceae*  *ge* | 5.09E-05 | 1.22E-05 | 1.03E-02 |
| *Fibrobacterota* | *Fibrobacteraceae* | *Fibrobacter* | 1.77E-04 | 2.86E-05 | 1.11E-02 |
| *Firmicutes* | *Aerococcaceae* | *Abiotrophia* | 1.55E-04 | 1.40E-04 | 2.72E-02 |
| *Firmicutes* | *Peptostreptococcaceae* | *Acetoanaerobium* | 6.49E-05 | 1.64E-05 | 3.91E-02 |
| *Firmicutes* | *Aerococcaceae* | *Aerococcaceae*  *unclassified* | 3.86E-05 | 5.41E-06 | 1.90E-02 |
| *Firmicutes* | *Bacillaceae* | *Bacillus* | 2.28E-03 | 9.31E-04 | 2.03E-02 |
| *Firmicutes* | *Lachnospiraceae* | *Blautia* | 5.34E-02 | 2.22E-02 | 4.28E-02 |
| *Firmicutes* | *Lachnospiraceae* | *Butyrivibrio* | 1.38E-05 | 1.14E-04 | 4.33E-02 |
| *Firmicutes* | *Lachnospiraceae* | *Cellulosilyticum* | 9.66E-05 | 8.87E-06 | 4.33E-02 |
| *Firmicutes* | *Desulfitobacteriaceae* | *Desulfosporosinus* | 3.88E-05 | 1.11E-05 | 1.88E-02 |
| *Firmicutes* | *Erysipelatoclostridiaceae* | *Erysipelatoclostridium* | 1.07E-02 | 3.71E-03 | 1.37E-02 |
| *Firmicutes* | *Erysipelotrichaceae* | *Erysipelotrichaceae*  *ge* | 4.34E-03 | 1.85E-03 | 3.34E-02 |
| *Firmicutes* | *Bacillaceae* | *Fictibacillus* | 3.43E-05 | 1.87E-05 | 4.33E-02 |
| *Firmicutes* | *Caloramatoraceae* | *Fonticella* | 5.35E-05 | 1.92E-05 | 4.46E-02 |
| *Firmicutes* | *Leuconostocaceae* | *Fructobacillus* | 6.60E-06 | 0.00E+00 | 4.33E-02 |
| *Firmicutes* | *Peptostreptococcaceae* | *Intestinibacter* | 5.75E-03 | 2.04E-03 | 1.83E-02 |
| *Firmicutes* | *Thermoactinomycetaceae* | *Laceyella* | 7.33E-05 | 0.00E+00 | 2.03E-02 |
| *Firmicutes* | *Lachnospiraceae* | *Lachnospiraceae*  *unclassified* | 7.36E-02 | 4.04E-02 | 3.89E-02 |
| *Firmicutes* | *Listeriaceae* | *Listeria* | 4.37E-04 | 1.07E-04 | 1.37E-02 |
| *Firmicutes* | *Oscillospiraceae* | *Oscillospiraceae ge* | 4.42E-05 | 0.00E+00 | 3.08E-02 |
| *Firmicutes* | *Paenibacillaceae* | *Paenibacillaceae*  *unclassified* | 8.64E-05 | 1.76E-05 | 5.43E-03 |
| *Firmicutes* | *Peptostreptococcaceae* | *Paeniclostridium* | 1.66E-03 | 2.21E-04 | 3.08E-02 |
| *Firmicutes* | *Peptostreptococcaceae* | *Peptostreptococcaceae*  *unclassified* | 1.52E-03 | 6.34E-04 | 4.99E-02 |
| *Firmicutes* | *Peptostreptococcaceae* | *Romboutsia* | 2.01E-02 | 6.97E-03 | 8.92E-03 |
| *Gemmatimonadota* | *AKAU4049 fa* | *AKAU4049 ge* | 7.58E-05 | 2.60E-05 | 3.29E-02 |
| *Gemmatimonadota* | *Longimicrobiaceae* | *Longimicrobiaceae*  *ge* | 1.05E-04 | 2.34E-05 | 3.08E-02 |
| *Gemmatimonadota* | *Longimicrobiaceae* | *Longimicrobiaceae*  *unclassified* | 1.90E-05 | 1.28E-06 | 2.37E-02 |
| *Gemmatimonadota* | *Gemmatimonadaceae* | *Roseisolibacter* | 1.42E-04 | 6.88E-05 | 1.11E-02 |
| *MBNT15* | *MBNT15 fa* | *MBNT15 ge* | 1.85E-04 | 8.53E-05 | 1.90E-02 |
| *Methylomirabilota* | *Methylomirabilaceae* | *Sh765B-TzT-35* | 6.00E-05 | 9.91E-06 | 1.77E-02 |
| *Methylomirabilota* | *Methylomirabilaceae* | *wb1-A12* | 2.50E-05 | 3.39E-06 | 3.91E-02 |
| *Myxococcota* | *bacteriap25 fa* | *bacteriap25 ge* | 6.45E-05 | 1.51E-05 | 3.23E-02 |
| *Myxococcota* | *BIrii41* | *BIrii41 ge* | 3.24E-04 | 6.07E-05 | 6.65E-03 |
| *Myxococcota* | *Haliangiaceae* | *Haliangium* | 6.71E-04 | 2.27E-04 | 1.89E-02 |
| *Myxococcota* | *Phaselicystidaceae* | *Phaselicystis* | 4.52E-05 | 1.23E-05 | 3.91E-02 |
| *Myxococcota* | *Sandaracinaceae* | *Sandaracinus* | 1.62E-04 | 8.27E-06 | 3.34E-02 |
| *Planctomycetota* | *OM190 fa* | *OM190 ge* | 2.35E-04 | 4.15E-05 | 3.08E-02 |
| *Proteobacteria* | *A21b* | *A21b ge* | 2.14E-04 | 6.44E-05 | 3.44E-02 |
| *Proteobacteria* | *Nitrincolaceae* | *Amphritea* | 6.68E-05 | 1.47E-05 | 8.46E-03 |
| *Proteobacteria* | *Diplorickettsiaceae* | *Aquicella* | 6.60E-05 | 2.03E-05 | 2.72E-02 |
| *Proteobacteria* | *Rhodocyclaceae* | *Azoarcus* | 1.31E-03 | 1.62E-04 | 3.69E-02 |
| *Proteobacteria* | *Pseudomonadaceae* | *Azotobacter* | 2.07E-05 | 1.83E-06 | 3.31E-02 |
| *Proteobacteria* | *Rhizobiaceae* | *Brucella* | 1.98E-04 | 4.97E-04 | 3.08E-02 |
| *Proteobacteria* | *Burkholderiaceae* | *Burkholderia-Caballeronia-Paraburkholderia* | 1.62E-03 | 9.98E-04 | 3.23E-02 |
| *Proteobacteria* | *Rhodocyclaceae* | *Candidatus Accumulibacter* | 2.49E-05 | 1.47E-05 | 3.66E-02 |
| *Proteobacteria* | *Acetobacteraceae* | *Craurococcus-Caldovatus* | 3.85E-05 | 1.28E-05 | 4.33E-02 |
| *Proteobacteria* | *Methylomonadaceae* | *Crenothrix* | 1.58E-05 | 2.04E-05 | 4.37E-02 |
| *Proteobacteria* | *Rhizobiaceae* | *Ensifer* | 8.27E-05 | 1.41E-05 | 1.90E-02 |
| *Proteobacteria* | *EPR3968-O8a-Bc78 fa* | *EPR3968-O8a-Bc78 ge* | 6.05E-05 | 2.10E-05 | 1.90E-02 |
| *Proteobacteria* | *Gallionellaceae* | *Gallionella* | 3.43E-05 | 8.81E-06 | 1.03E-02 |
| *Proteobacteria* | *Acetobacteraceae* | *Gluconobacter* | 2.80E-05 | 8.05E-06 | 2.37E-02 |
| *Proteobacteria* | *Halomonadaceae* | *Halovibrio* | 5.24E-05 | 0.00E+00 | 3.08E-02 |
| *Proteobacteria* | *HOC36 fa* | *HOC36 ge* | 3.25E-05 | 2.06E-06 | 1.37E-02 |
| *Proteobacteria* | *Hydrogenophilaceae* | *Hydrogenophilus* | 3.89E-04 | 2.42E-03 | 2.10E-02 |
| *Proteobacteria* | *Immundisolibacteraceae* | *Immundisolibacter* | 8.16E-05 | 4.10E-05 | 2.63E-02 |
| *Proteobacteria* | *Comamonadaceae* | *Inhella* | 1.17E-05 | 0.00E+00 | 3.08E-02 |
| *Proteobacteria* | *JG36-TzT-191 fa* | *JG36-TzT-191 ge* | 1.28E-04 | 2.86E-05 | 3.08E-02 |
| *Proteobacteria* | *JTB23 fa* | *JTB23 ge* | 7.24E-05 | 8.16E-06 | 3.08E-02 |
| *Proteobacteria* | *Kiloniellaceae* | *Limibacillus* | 9.96E-06 | 0.00E+00 | 4.33E-02 |
| *Proteobacteria* | *Xanthomonadaceae* | *Lysobacter* | 5.15E-04 | 2.57E-04 | 4.08E-02 |
| *Proteobacteria* | *Nitrincolaceae* | *Marinobacterium* | 1.60E-04 | 6.46E-05 | 1.72E-02 |
| *Proteobacteria* | *Oxalobacteraceae* | *Massilia* | 3.76E-03 | 1.18E-03 | 6.70E-03 |
| *Proteobacteria* | *MBMPE27 fa* | *MBMPE27 ge* | 3.99E-05 | 6.75E-06 | 1.59E-02 |
| *Proteobacteria* | *Methylomonadaceae* | *Methylobacter* | 1.50E-05 | 6.59E-06 | 3.47E-02 |
| *Proteobacteria* | *Micropepsaceae* | *Micropepsaceae*  *unclassified* | 1.43E-04 | 4.29E-05 | 8.92E-03 |
| *Proteobacteria* | *Nitrincolaceae* | *Motiliproteus* | 1.24E-05 | 0.00E+00 | 4.33E-02 |
| *Proteobacteria* | *Solimonadaceae* | *Panacagrimonas* | 3.70E-05 | 8.13E-06 | 1.89E-02 |
| *Proteobacteria* | *PLTA13 fa* | *PLTA13 ge* | 1.46E-04 | 8.41E-05 | 2.03E-02 |
| *Proteobacteria* | *Oxalobacteraceae* | *Pseudoduganella* | 1.07E-03 | 1.43E-04 | 1.04E-02 |
| *Proteobacteria* | *Xanthomonadaceae* | *Pseudoxanthomonas* | 2.42E-04 | 8.96E-05 | 4.41E-02 |
| *Proteobacteria* | *R7C24 fa* | *R7C24 ge* | 6.48E-05 | 3.52E-05 | 1.07E-02 |
| *Proteobacteria* | *Micropepsaceae* | *Rhizomicrobium* | 3.14E-05 | 2.07E-06 | 3.15E-02 |
| *Proteobacteria* | *Rhodanobacteraceae* | *Rhodanobacter* | 6.79E-04 | 3.96E-04 | 3.34E-02 |
| *Proteobacteria* | *Xanthobacteraceae* | *Rhodoplanes* | 5.03E-05 | 7.66E-06 | 2.22E-02 |
| *Proteobacteria* | *Rhodobacteraceae* | *Rhodovulum* | 0.00E+00 | 1.32E-05 | 4.33E-02 |
| *Proteobacteria* | *Beijerinckiaceae* | *Roseiarcus* | 1.47E-04 | 3.91E-05 | 3.47E-02 |
| *Proteobacteria* | *Steroidobacteraceae* | *Steroidobacter* | 2.50E-04 | 1.10E-04 | 4.28E-02 |
| *Proteobacteria* | *Steroidobacteraceae* | *Steroidobacteraceae*  *unclassified* | 6.49E-05 | 1.60E-05 | 2.91E-02 |
| *Proteobacteria* | *Succinivibrionaceae* | *Succinivibrio* | 6.25E-05 | 2.56E-05 | 4.20E-02 |
| *Proteobacteria* | *Sulfuricellaceae* | *Sulfuriferula* | 5.46E-05 | 0.00E+00 | 1.03E-02 |
| *Proteobacteria* | *Chromatiaceae* | *Thiocapsa* | 1.34E-05 | 0.00E+00 | 2.03E-02 |
| *Proteobacteria* | *TRA3-20* | *TRA3-20 ge* | 3.66E-04 | 1.23E-04 | 1.37E-02 |
| *Proteobacteria* | *Oxalobacteraceae* | *Undibacterium* | 4.96E-05 | 4.68E-06 | 6.65E-03 |
| *Proteobacteria* | *Nitrosococcaceae* | *wb1-P19* | 1.56E-05 | 0.00E+00 | 3.08E-02 |
| *Proteobacteria* | *WD260 fa* | *WD260 ge* | 5.56E-04 | 1.23E-04 | 6.60E-03 |
| *Proteobacteria* | *Xanthomonadaceae* | *Xanthomonas* | 1.78E-05 | 4.67E-04 | 2.65E-02 |
| *RCP2-54* | *RCP2-54 fa* | *RCP2-54 ge* | 5.60E-04 | 1.04E-04 | 6.65E-03 |
| *Verrucomicrobiota* | *Opitutaceae* | *Lacunisphaera* | 7.16E-04 | 4.73E-05 | 1.89E-02 |
| *Verrucomicrobiota* | *P.palmC41 fa* | *P.palmC41 ge* | 0.00E+00 | 3.99E-05 | 3.08E-02 |
| *Verrucomicrobiota* | *S-BQ2-57 soil*  *group fa* | *S-BQ2-57 soil group ge* | 2.09E-05 | 3.11E-07 | 1.62E-02 |

**Supplementary Table S5**. List of significantly different genera in the gut microbiome of *Nephrops norvegicus* between *'Hematodinium*-free' (Negative by BCM, PM, PCR, and Histology; n=22) and *Hematodinium* positive (Positive by BCM, Pleopod Stages 3 and 4; n=22) (Wilcoxon rank-sum, *p* < 0.05).

| Taxonomy | | | Log 10 mean relative abundance | | Adjusted *p*-value |
| --- | --- | --- | --- | --- | --- |
| Phylum | **Family** | **Genus** | **Negative** | **Positive** |  |
| *Acidobacteriota* | *Vicinamibacterales unclassified* | *Vicinamibacterales*  *unclassified* | 4.21E-06 | 2.93E-05 | 1.45E-02 |
| *Actinobacteriota* | *Bifidobacteriaceae* | *Bifidobacterium* | 3.68E-04 | 4.04E-04 | 2.13E-02 |
| *Actinobacteriota* | *Actinomarinaceae* | *Candidatus*  *Actinomarina* | 1.48E-06 | 1.98E-05 | 1.82E-02 |
| *Actinobacteriota* | *Solirubrobacteraceae* | *Conexibacter* | 1.09E-05 | 6.06E-05 | 1.95E-02 |
| *Actinobacteriota* | *Corynebacteriaceae* | *Corynebacterium* | 4.12E-05 | 3.03E-04 | 2.85E-02 |
| *Actinobacteriota* | *Propionibacteriaceae* | *Cutibacterium* | 5.99E-06 | 3.93E-05 | 2.76E-02 |
| *Actinobacteriota* | *Nocardioidaceae* | *Marmoricola* | 4.34E-06 | 2.04E-05 | 3.57E-02 |
| *Actinobacteriota* | *Micrococcaceae* | *Micrococcus* | 7.92E-06 | 3.27E-05 | 3.75E-02 |
| *Actinobacteriota* | *OPB41 fa* | *OPB41 ge* | 6.29E-06 | 4.66E-05 | 4.29E-02 |
| *Actinobacteriota* | *Propionibacteriaceae* | *Propionibacteriaceae*  *unclassified* | 0.00E+00 | 1.69E-05 | 4.62E-03 |
| *Actinobacteriota* | *Micrococcaceae* | *Pseudarthrobacter* | 1.38E-05 | 1.40E-04 | 1.74E-02 |
| *Actinobacteriota* | *Micrococcaceae* | *Rothia* | 1.40E-05 | 4.67E-05 | 2.85E-02 |
| *Bacteroidota* | *Rikenellaceae* | *Alistipes* | 9.66E-05 | 2.75E-04 | 2.00E-02 |
| *Bacteroidota* | *Prevotellaceae* | *Alloprevotella* | 1.10E-05 | 9.06E-05 | 2.11E-02 |
| *Bacteroidota* | *Bacteroidaceae* | *Bacteroides* | 2.97E-04 | 1.10E-03 | 4.62E-03 |
| *Bacteroidota* | *Bacteroidetes vadinHA17* | *Bacteroidetes*  *vadinHA17 ge* | 0.00E+00 | 1.43E-05 | 2.00E-02 |
| *Bacteroidota* | *Chitinophagaceae* | *Chitinophagaceae*  *unclassified* | 9.66E-06 | 3.98E-05 | 4.54E-02 |
| *Bacteroidota* | *env.OPS 17* | *env.OPS 17 ge* | 0.00E+00 | 5.88E-06 | 2.00E-02 |
| *Bacteroidota* | *Tannerellaceae* | *Macellibacteroides* | 7.03E-07 | 2.98E-05 | 2.63E-02 |
| *Bacteroidota* | *Muribaculaceae* | *Muribaculaceae*  *ge* | 3.99E-03 | 1.08E-02 | 4.62E-03 |
| *Bacteroidota* | *Muribaculaceae* | *Muribaculaceae*  *unclassified* | 1.70E-05 | 9.07E-05 | 2.00E-02 |
| *Bacteroidota* | *NS9 marine group* | *NS9 marine group ge* | 6.62E-07 | 1.94E-05 | 1.71E-02 |
| *Bacteroidota* | *Tannerellaceae* | *Parabacteroides* | 6.51E-05 | 2.39E-04 | 5.82E-03 |
| *Bacteroidota* | *Prevotellaceae* | *Prevotella* | 5.27E-05 | 1.53E-04 | 4.29E-02 |
| *Bacteroidota* | *Prevotellaceae* | *Prevotellaceae*  *unclassified* | 3.51E-05 | 1.60E-04 | 1.26E-02 |
| *Bacteroidota* | *Rikenellaceae* | *Rikenellaceae*  *RC9 gut group* | 2.05E-05 | 7.24E-05 | 4.83E-02 |
| *Bacteroidota* | *Tannerellaceae* | *Tannerellaceae*  *unclassified* | 1.56E-05 | 5.58E-05 | 4.19E-02 |
| *Campilobacterota* | *Helicobacteraceae* | *Helicobacter* | 9.94E-05 | 2.98E-04 | 2.97E-02 |
| *Desulfobacterota* | *Desulfocapsaceae* | *Desulforhopalus* | 9.68E-03 | 2.32E-03 | 4.04E-02 |
| *Desulfobacterota* | *Desulfovibrionaceae* | *Desulfovibrionaceae*  *unclassified* | 1.16E-03 | 4.27E-04 | 2.00E-02 |
| *Firmicutes* | *Lachnospiraceae* | *A2* | 0.00E+00 | 8.34E-06 | 2.00E-02 |
| *Firmicutes* | *Lachnospiraceae* | *Anaerostipes* | 1.09E-05 | 4.81E-05 | 2.96E-02 |
| *Firmicutes* | *Anaerovoracaceae* | *Anaerovorax* | 0.00E+00 | 1.04E-05 | 2.96E-02 |
| *Firmicutes* | *Bacillaceae* | *Bacillaceae*  *unclassified* | 3.58E-05 | 1.64E-04 | 2.13E-02 |
| *Firmicutes* | *Clostridia UCG-014*  *fa* | *Clostridia*  *UCG-014 ge* | 8.49E-05 | 2.79E-04 | 9.24E-03 |
| *Firmicutes* | *Clostridia unclassified* | *Clostridia*  *unclassified* | 5.88E-05 | 1.46E-04 | 1.04E-02 |
| *Firmicutes* | *Clostridia vadinBB60 group fa* | *Clostridia*  *vadinBB60 group ge* | 9.38E-06 | 2.60E-05 | 2.97E-02 |
| *Firmicutes* | *Clostridiales unclassified* | *Clostridiales*  *unclassified* | 1.38E-06 | 2.24E-05 | 3.22E-02 |
| *Firmicutes* | *Lachnospiraceae* | *Coprococcus* | 9.03E-06 | 3.95E-05 | 3.13E-02 |
| *Firmicutes* | *Desulfitobacteriaceae* | *Desulfitobacterium* | 0.00E+00 | 2.06E-05 | 4.60E-02 |
| *Firmicutes* | *Erysipelotrichaceae* | *Dubosiella* | 3.11E-04 | 9.47E-04 | 6.17E-03 |
| *Firmicutes* | *Erysipelotrichaceae* | *Faecalibaculum* | 8.54E-06 | 3.42E-05 | 3.22E-02 |
| *Firmicutes* | *Firmicutes unclassified* | *Firmicutes*  *unclassified* | 1.01E-04 | 2.66E-04 | 3.57E-02 |
| *Firmicutes* | *Hungateiclostridiaceae* | *Hungateiclostridiaceae*  *unclassified* | 3.69E-06 | 5.15E-05 | 2.17E-02 |
| *Firmicutes* | *Limnochordia fa* | *Hydrogenispora* | 7.73E-07 | 1.67E-05 | 4.29E-02 |
| *Firmicutes* | *Erysipelotrichaceae* | *Ileibacterium* | 6.63E-07 | 3.01E-05 | 2.13E-02 |
| *Firmicutes* | *Lachnospiraceae* | *Lachnospira* | 8.67E-06 | 5.88E-05 | 1.45E-02 |
| *Firmicutes* | *Lachnospiraceae* | *Lachnospiraceae*  *ge* | 5.23E-05 | 2.49E-04 | 2.00E-02 |
| *Firmicutes* | *Lachnospiraceae* | *Lachnospiraceae*  *NK4A136 group* | 1.32E-04 | 3.78E-04 | 4.28E-02 |
| *Firmicutes* | *Lactobacillales*  *unclassified* | *Lactobacillales*  *unclassified* | 5.99E-05 | 1.82E-04 | 1.45E-02 |
| *Firmicutes* | *Lactobacillaceae* | *Lactobacillus* | 4.98E-04 | 2.63E-03 | 4.34E-02 |
| *Firmicutes* | *Monoglobaceae* | *Monoglobus* | 7.76E-06 | 2.93E-05 | 3.75E-02 |
| *Firmicutes* | *Oscillospiraceae* | *NK4A214 group* | 1.80E-05 | 6.31E-05 | 9.15E-03 |
| *Firmicutes* | *Oscillospiraceae* | *Oscillospiraceae*  *unclassified* | 3.32E-05 | 1.86E-04 | 7.98E-03 |
| *Firmicutes* | *Oscillospirales fa* | *Oscillospirales ge* | 2.96E-05 | 1.02E-04 | 2.47E-02 |
| *Firmicutes* | *Oscillospirales*  *unclassified* | *Oscillospirales*  *unclassified* | 1.11E-05 | 5.67E-05 | 2.00E-02 |
| *Firmicutes* | *Oxobacteraceae* | *Oxobacter* | 0.00E+00 | 1.37E-05 | 4.60E-02 |
| *Firmicutes* | *Peptostreptococcaceae* | *Peptoclostridium* | 3.94E-04 | 2.84E-05 | 3.13E-02 |
| *Firmicutes* | *Sedimentibacteraceae* | *Sedimentibacter* | 3.38E-07 | 1.55E-05 | 2.17E-02 |
| *Firmicutes* | *Acidaminococcaceae* | *Succiniclasticum* | 0.00E+00 | 1.02E-05 | 4.60E-02 |
| *Firmicutes* | *Erysipelotrichaceae* | *Turicibacter* | 7.95E-05 | 2.09E-04 | 2.05E-02 |
| *Firmicutes* | *UCG-010* | *UCG-010 ge* | 8.94E-06 | 5.02E-05 | 1.93E-02 |
| *Firmicutes* | *Veillonellaceae* | *Veillonella* | 2.05E-05 | 1.48E-04 | 2.17E-02 |
| *Firmicutes* | *Lachnospiraceae* | *XBB1006* | 3.29E-07 | 3.04E-05 | 2.17E-02 |
| *Fusobacteriota* | *Fusobacteriaceae* | *Fusobacterium* | 1.80E-05 | 1.08E-04 | 4.62E-03 |
| *Proteobacteria* | *Acetobacteraceae* | *Acetobacteraceae*  *unclassified* | 8.43E-06 | 3.52E-05 | 2.13E-02 |
| *Proteobacteria* | *Moraxellaceae* | *Acinetobacter* | 2.04E-04 | 3.35E-04 | 1.45E-02 |
| *Proteobacteria* | *Alcaligenaceae* | *Alcaligenaceae*  *unclassified* | 2.00E-05 | 5.80E-05 | 4.28E-02 |
| *Proteobacteria* | *Alcaligenaceae* | *Alcaligenes* | 3.47E-05 | 1.88E-04 | 4.62E-03 |
| *Proteobacteria* | *Burkholderiaceae* | *Burkholderia*  *Caballeronia*  *Paraburkholderia* | 2.07E-05 | 6.46E-05 | 4.28E-02 |
| *Proteobacteria* | *Moraxellaceae* | *Enhydrobacter* | 6.93E-06 | 4.57E-05 | 2.05E-02 |
| *Proteobacteria* | *Rhodobacteraceae* | *HIMB11* | 1.74E-05 | 1.29E-04 | 2.00E-02 |
| *Proteobacteria* | *Hydrogenophilaceae* | *Hydrogenophilus* | 3.91E-05 | 8.68E-05 | 1.26E-02 |
| *Proteobacteria* | *Rhodobacteraceae* | *Marivivens* | 8.28E-07 | 3.33E-05 | 1.26E-02 |
| *Proteobacteria* | *Oxalobacteraceae* | *Massilia* | 1.15E-05 | 5.16E-05 | 2.00E-02 |
| *Proteobacteria* | *Beijerinckiaceae* | *Methylobacterium-*  *Methylorubrum* | 3.61E-05 | 1.66E-03 | 2.25E-02 |
| *Proteobacteria* | *Sphingomonadaceae* | *Novosphingobium* | 4.12E-06 | 3.18E-05 | 2.00E-02 |
| *Proteobacteria* | *Oxalobacteraceae* | *Oxalobacteraceae*  *unclassified* | 9.20E-06 | 3.73E-05 | 2.00E-02 |
| *Proteobacteria* | *Sphingomonadaceae* | *Qipengyuania* | 0.00E+00 | 1.14E-05 | 1.54E-02 |
| *Proteobacteria* | *Rhizobiales*  *unclassified* | *Rhizobiales*  *unclassified* | 1.04E-04 | 5.41E-04 | 1.71E-02 |
| *Proteobacteria* | *Acetobacteraceae* | *Roseomonas* | 0.00E+00 | 1.23E-05 | 2.00E-02 |
| *Proteobacteria* | *Porticoccaceae* | *SAR92 clade* | 6.61E-07 | 2.69E-05 | 3.85E-02 |
| *Proteobacteria* | *SC-I-84* | *SC-I-84 ge* | 9.14E-06 | 4.45E-05 | 1.26E-02 |
| *Proteobacteria* | *Sphingomonadaceae* | *Sphingomonadaceae*  *unclassified* | 2.61E-05 | 1.20E-04 | 4.62E-03 |
| *Verrucomicrobiota* | *Akkermansiaceae* | *Akkermansia* | 3.78E-05 | 1.41E-04 | 8.06E-03 |
| *Verrucomicrobiota* | *Lentisphaeria unclassified* | *Lentisphaeria*  *unclassified* | 1.03E-03 | 6.15E-04 | 2.19E-02 |
| *Verrucomicrobiota* | *P.palmC41 fa* | *P.palmC41 ge* | 1.01E-01 | 4.47E-02 | 1.26E-02 |
| *WS4* | *WS4 fa* | *WS4 ge* | 0.00E+00 | 2.73E-05 | 4.60E-02 |
