# Supplementary material for: Microbiome dynamics associated with Hematodinium sp. infection in Norway lobster (Nephrops norvegicus)
Source: Anim Microbiome. 2025 Jun 13;7:62. doi: 10.1186/s42523-025-00416-w (PMC12164141; doi:10.1186/s42523-025-00416-w)

**Supplementary Figure Legends**

**Supplementary Figure S1.** Progression of *Hematodinium* sp. infection in gill tissue: stages 0 to 4 based on the pleopod method (Left to Right) (Scale 100μm).

**Supplementary Figure S2.** Progression of *Hematodinium* sp. infection in the heart: stages 0 to 4 based on the pleopod method (left to right). In stage 0 of infection (top-left image), the haemal spaces exhibit a dense population of hemocytes, and no parasites are observed. As the infection progresses, there is a gradual decrease in hemocyte count (Scale 100μm).

**Supplementary Figure S3.** Progression of *Hematodinium* sp. infection in the hepatopancreas: stages 0 to 4 based on the pleopod method (left to right) (Scale 100μm).

**Supplementary Figure S4.** Progression of *Hematodinium* sp. infection in the muscle: stages 0 to 4 based on the pleopod method (left to right) (Scale 100μm).

**Supplementary Figure S5.** Progression of *Hematodinium* sp. infection in the female gonad: stages 0 to 4 based on the pleopod method (left to right) (Scale 100μm).

**Supplementary Figure S6.** Progression of *Hematodinium* sp. infection in the male gonad: stages 0 to 4 based on the pleopod method (left to right) (Scale 100μm).


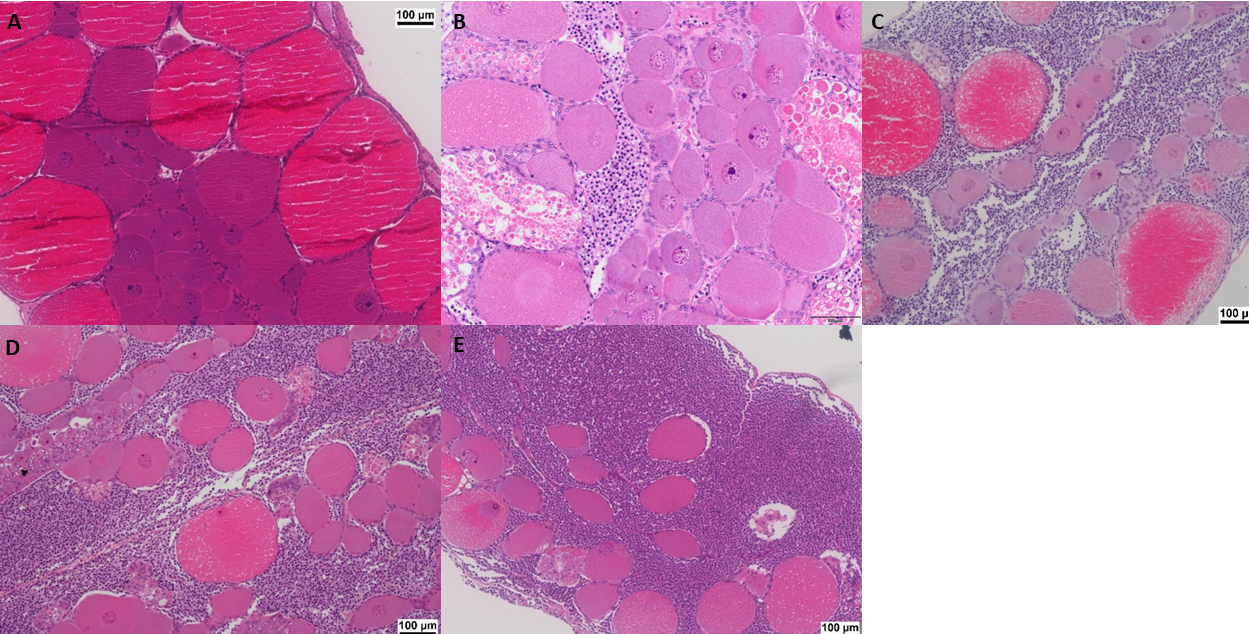

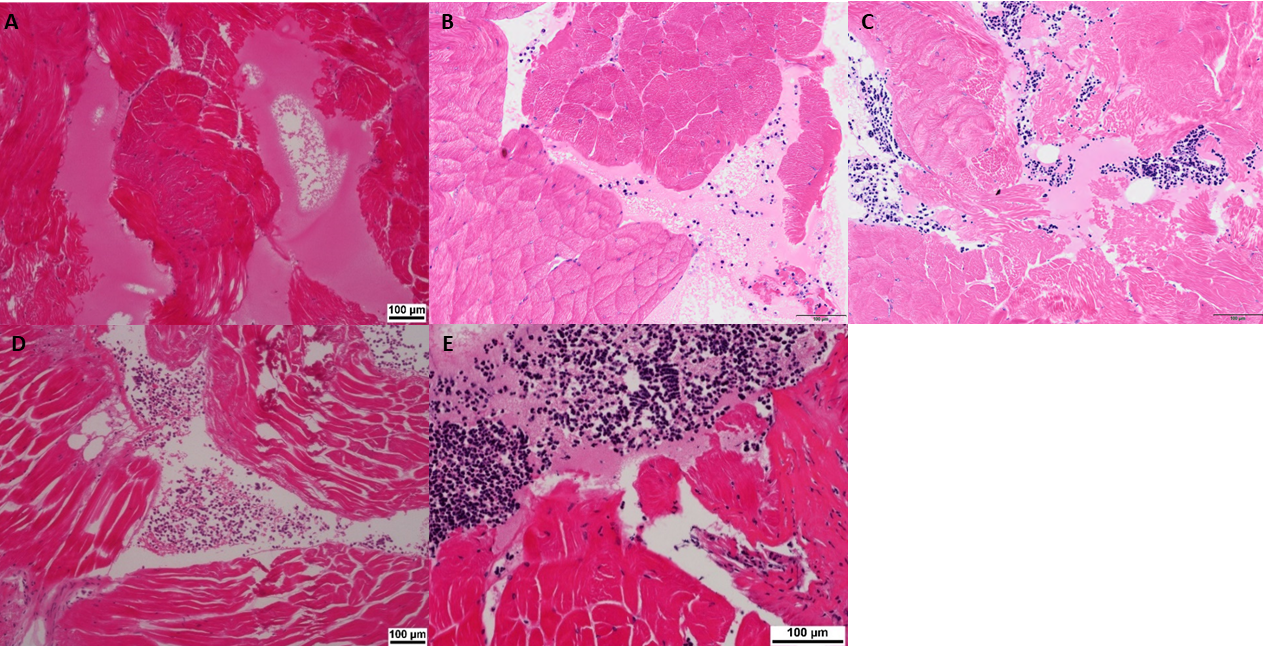

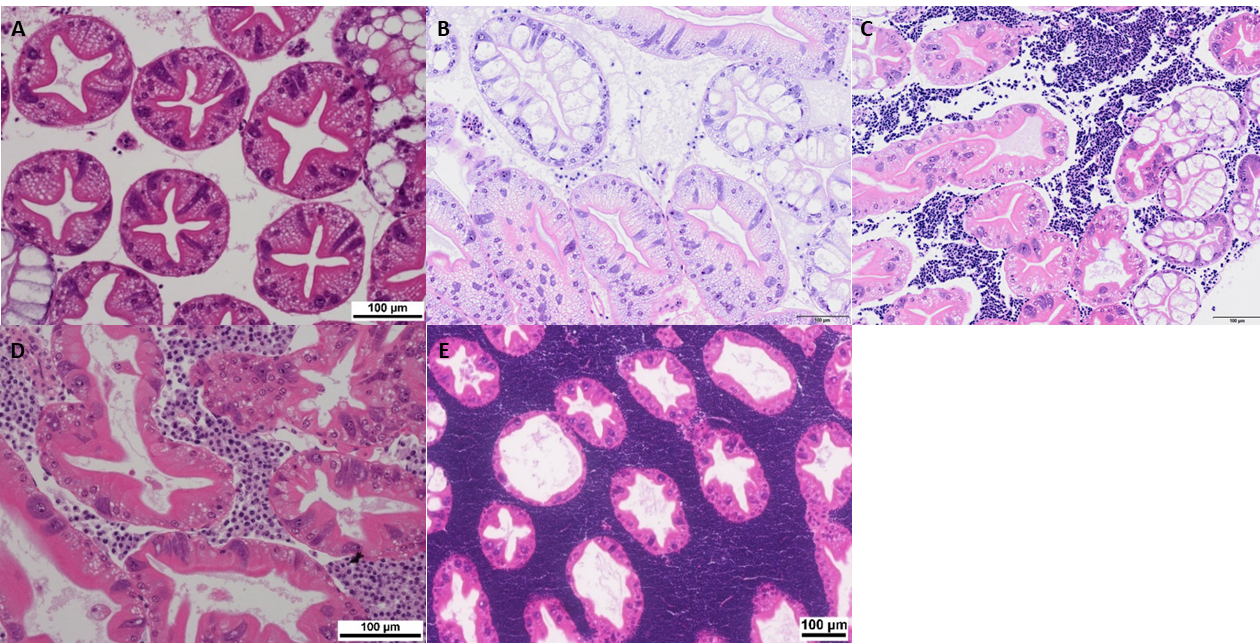

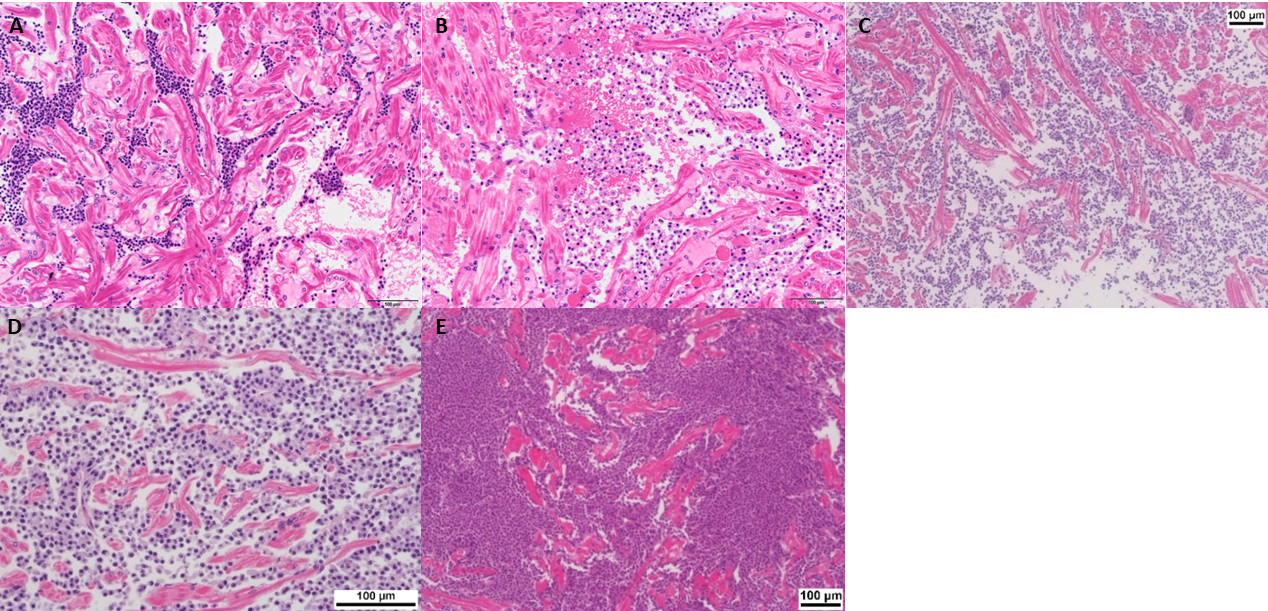

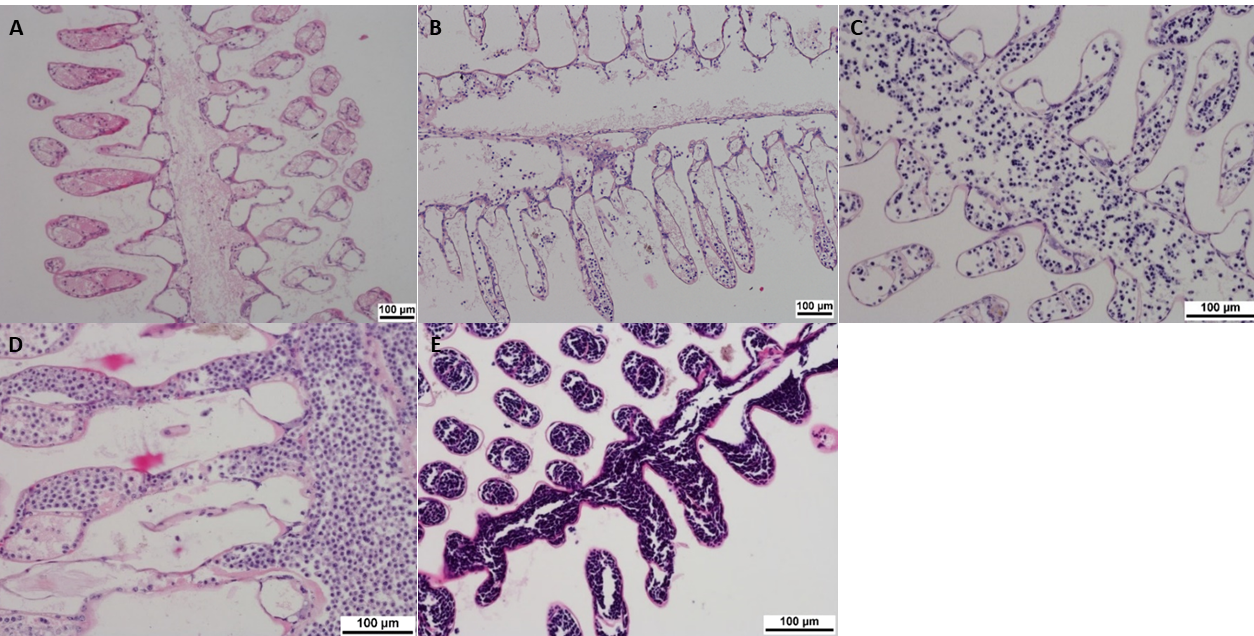

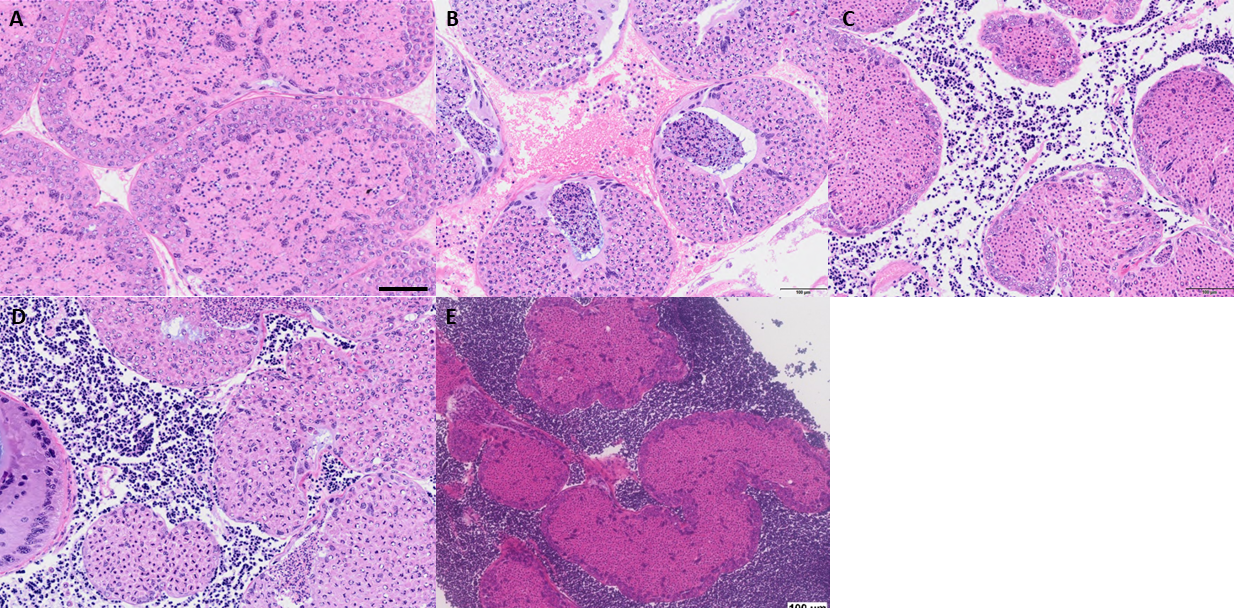

Supplement: Supplementary file 2 — Additional file 2. [file 42523_2025_416_MOESM2_ESM.docx]
